# Nonlinearity-enabled higher-order exceptional singularities with ultra-enhanced signal-to-noise ratio

**DOI:** 10.1093/nsr/nwac259

**Published:** 2022-11-16

**Authors:** Kai Bai, Liang Fang, Tian-Rui Liu, Jia-Zheng Li, Duanduan Wan, Meng Xiao

**Affiliations:** Key Laboratory of Artificial Micro- and Nano-structures of Ministry of Education and School of Physics and Technology, Wuhan University, Wuhan 430072, China; Key Laboratory of Artificial Micro- and Nano-structures of Ministry of Education and School of Physics and Technology, Wuhan University, Wuhan 430072, China; Key Laboratory of Artificial Micro- and Nano-structures of Ministry of Education and School of Physics and Technology, Wuhan University, Wuhan 430072, China; Key Laboratory of Artificial Micro- and Nano-structures of Ministry of Education and School of Physics and Technology, Wuhan University, Wuhan 430072, China; Key Laboratory of Artificial Micro- and Nano-structures of Ministry of Education and School of Physics and Technology, Wuhan University, Wuhan 430072, China; Key Laboratory of Artificial Micro- and Nano-structures of Ministry of Education and School of Physics and Technology, Wuhan University, Wuhan 430072, China; Wuhan Institute of Quantum Technology, Wuhan 430206, China

**Keywords:** higher-order exceptional point, nonlinearity, ultra-enhanced signal-to-noise ratio

## Abstract

Higher-order exceptional points (HOEPs) with extraordinary responsivity are expected to exhibit a vastly improved performance in detection-related applications. However, over the past few years, such an approach has been questioned due to several potential drawbacks, including the stringent parameter requirements, fundamental resolution limits and noise. Here, exploring the consequence of nonlinear gain saturation in exceptional singularities of non-Hermitian systems, we offer a feasible scheme to overcome all the above difficulties. We provide a simple and intuitive example by demonstrating with both theory and circuit experiments an ‘exceptional nexus’ (‘EX’), a HOEP with an ultra-enhanced signal-to-noise ratio (SNR), in only two coupled resonators with the aid of nonlinear gain. The tedious parameter tuning in a six-dimensional hyper-dimensional space is reduced to two dimensions. The feedback mechanism of nonlinear saturable gain can give a solution to the ongoing debate on the SNR of EPs in other linear systems. Our findings advance the fundamental understanding of the peculiar topology of nonlinear non-Hermitian systems, significantly reduce the practical difficulty in EP sensing and possibly open new avenues for applications.

## Teaser

Exploring the nonlinear gain saturation ends a long-lasting debate about non-enhanced signal-to-noise ratio in the proximity of exceptional singularities, and thus paves the way for promising ultrasensitive sensing devices.

## INTRODUCTION

The physical dimension of a system is of vital importance in topological physics, and a higher dimension enables diverse and complex topology. The interplay of the dimension and topology of a non-Hermitian system is even more fascinating, and even a one-dimensional single band can exhibit nontrivial topology as the complex plane is introduced [1]. The recent advances in non-Hermitian physics are underlain by the ‘exceptional’ constituents [2–11]. There are exceptional points (EPs) [12,13], rings [14,15], surfaces [16], bulk exceptional arcs (EAs) [17] and exceptional nexuses (EXs) [18] as a manifestation of different physical dimensions. Higher physical dimensions enable the forming of HOEPs, which then lead to the enhancement of light-matter interactions[19,20], sensing [21] and mechanical damping and spring stiffness in optomechanics [22]. Here, we provide a new approach to realizing HOEPs with the aid of nonlinearity and demonstrate that a three-dimensional (3D) ‘EX’ can be achieved even within two cavities.

The integration of nonlinearity with topology in Hermitian systems brings fruitful novel phenomena [23–30], while research on the exceptional singularities of nonlinear non-Hermitian topological physics is rare [27–32]. In stark contrast, nonlinearity is natural in non-Hermitian systems, especially when the gain is introduced. From the energy consideration, one needs to consider a more realistic nonlinear saturable gain that then leads to beneficial applications such as wireless energy transfer [33] and optical bistability [34]. To date, most works on non-Hermitian systems have been restricted to the linear region. Our work provides a simple and intuitive example and shows how the nonlinear saturable gain can introduce a hidden dimension that further lead to an EX. Different from previous works [23–30], such physics discussed here requires an additional dimension besides its apparent physical dimension, which thus cannot be captured within the dimension of the linear region. Our work points to the possibility of exploring higher-dimensional (≥3) physics in a lower-dimensional (=2) system with the aid of nonlinearity.

A HOEP, such as an EX, is expected to exhibit the potential to enhance the performance of the sensor [21,35,36]. However, the possible drawbacks [5,21,35,37–45], including the stringent parameter requirements, fundamental resolution limits and noise, render the implementation of HOEPs within the existing schemes. Here in our work, these seeming incompatibilities can be naturally reconciliated: the exquisite dimension correlation in our system offers us unique advantages in utilizing the exceptional features of non-Hermitian systems. The ‘EX’ condition can be reached by changing only very limited parameters. (Reduce to two parameters from six parameters for linear EXs.) Meanwhile, some fundamental resolution limits and noise enhanced by the nonorthogonality of states near the EPs can be surpassed thanks to the feedback mechanism of the nonlinear saturable gain. To be more specific, different from linear systems wherein the noise fluctuations grow with time [37,45], our system will reach and stay near only one stable state almost forever. In addition, when approaching the ‘EX’ point, the divergence of the eigenvalue susceptibility surpasses the amplification of noise (linewidth of spectra) originated from the coalescence of the eigenstates [38], and thus the SNR is dramatically improved.

## RESULTS

### Tight-binding model

**Figure 1.**
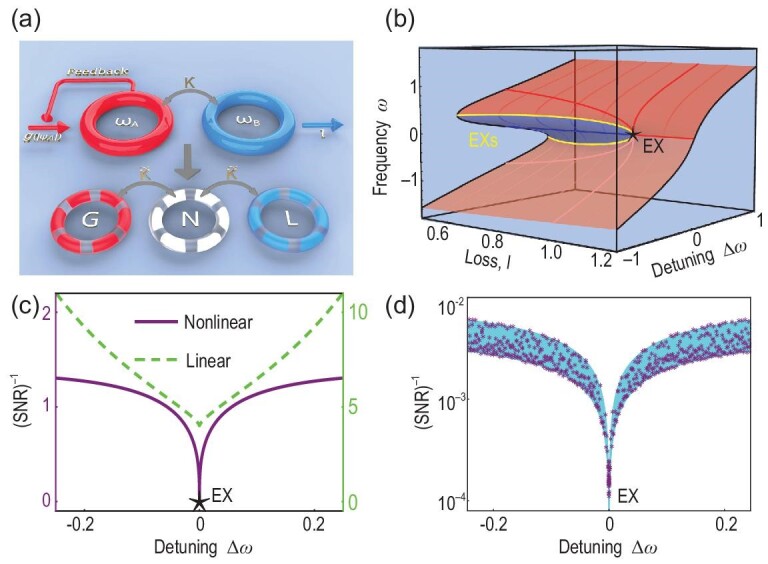
(a) The upper panel shows two coupled resonators. The left resonator (red) has resonance frequency ω_*A*_ and nonlinear saturable gain *g*(|ψ_*A*_|) (see the discussions in Section 8 of the online supplementary material), and the other resonator (blue) has resonance frequency ω_*B*_ and linear loss. Such a nonlinear non-Hermitian system can be mapped into a PT symmetric three-resonator system as shown in the lower panel with linear gain (G) and neutral (N) and loss (L) resonators. (b) Steady-state solution of (1) with κ=1, ω_*B*_ = 0 and all the other parameters normalized by κ. The red and blue regions represent stable and unstable states, respectively. The yellow lines represent EAs that are also boundaries of the stable and unstable states. The two yellow lines merge at an EX (black star), where two stable states and one unstable state coalesce. (c) (SNR)^−1^ as a function of the detuning for a linear scheme (green dashed line), and a nonlinear saturable scheme (purple line). Here sensing is viewed as a scattering process of the input photon. The linear Hamiltonian is provided in Equation (S24) of the online supplementary material. (d) Purple asterisks show the SNR retrieved from the system dynamics and the cyan background represents the line width limit introduced by the finite recording time. Details are provided in Section 4 of the online supplementary material.

Our system consists of two coupled resonators with nonlinear gain (red) and loss (blue) as sketched in the upper panel of Fig. 1(a). The system dynamics satisfy the nonlinear equation


(1) \begin{eqnarray*} && i\frac{d}{dt} \left(\begin{array}{c}\psi _{A}\\ \psi _{B} \end{array}\right) \\ && = \left(\begin{array}{c}\omega _{A}+ig(|\psi _{A}|)+i\zeta \xi _{g}(t) \quad\quad \kappa \\ \quad\quad\quad\quad\quad\kappa \quad\quad\quad\quad\quad\quad \omega _{B}-il \end{array}\!\! \right) \left(\begin{array}{c}\psi _{A}\\ \psi _{B} \end{array}\!\right) \\ &&\quad +\, D \left(\begin{array}{c}\xi _{e}(t)\\ 0 \end{array}\right), \end{eqnarray*}

where ω_*A*_ (ω_*B*_) is the resonant frequency of the left (right) resonator, ψ_*A, B*_ represents the corresponding field amplitude that is defined such that |ψ_*A, B*_|^2^ is the energy stored in each resonator, κ denotes the strength of coupling, *l* denotes the loss, *g* represents the gain, which is assumed to depend on |ψ_*A*_|. For later discussions, we also include the Gaussian white noise functions ξ_*g*_(*t*) and ξ_*e*_(*t*) with mean 0 and corresponding standard deviations ζ and *D*, respectively [46]. Here, ξ_*g*_(*t*) is introduced by the imperfect feedback mechanism and instability of gain, ξ_*e*_(*t*) is from white noise of the environment and assumed to be only in the left resonator for simplicity, and the phenomena are similar if both resonators have white noise. The steady-state solutions without noise (*D* = ζ = 0) satisfy


(2) \begin{eqnarray*} &&(\omega -\omega _{A})(\omega -\omega _{B})^{2} +(\omega -\omega _{A})l^{2} \\ &&\quad -\,\kappa ^{2}(\omega -\omega _{B}) = 0 \end{eqnarray*}

and


(3) \begin{eqnarray*} g_{s}=\frac{\omega _{A}-\omega }{\omega _{B}-\omega }l. \end{eqnarray*}

Here noting that the magnitude of gain *g* depends on the field amplitude, (3) being satisfied at *g* = *g_s_* can be achieved by adjusting the wave function |ψ_*A*_|. Figure 1(b) shows the real ω solution of (2) versus loss *l* and detuning Δ_ω_ ≡ ω_*A*_ − ω_*B*_, where we set κ = 1 and ω_*B*_ = 0. Here, for simplicity, all the parameters are normalized by κ and become dimensionless. At zero detuning, there are three states when *l* < κ, as denoted by the bold (light) red and blue lines, and these three states coalesce into one at *l* = κ, as marked by the black star. Meanwhile, over a finite detuning range, these three states are preserved until two of them coalesce at the yellow lines. Later in the text, we prove that the point marked by a black star is a nonlinear ‘EX’. As shown in Fig. 1(c), such an ‘EX’ exhibits a diverging SNR (purple line) for steady states in contrast to another EX from a linear system where the SNR is finite (green dashed line). If we further consider the system dynamics wherein the feedback mechanism is involved, the actual SNR is better, as shown in Fig. 1(d). Note that the specific form of gain is irrelevant for the physics discussed herein provided that (3) can be satisfied.

**Figure 2.**
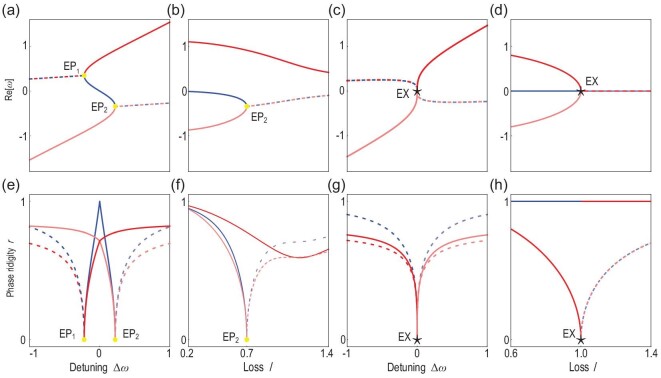
(a–d) Real part of the solutions of (2) versus the detuning and loss, where the red and blue solid lines represent stable and unstable steady states, respectively, and the red and blue dashed lines represent complex solutions of (2) with positive and negative imaginary parts, respectively. In (a) *l* = 0.7, in (b) Δ_ω_ = 0.22, in (c) *l* = 1, in (d) Δ_ω_ = 0, and all the other parameters are the same as in Fig. 1(b). (e–h) Phase rigidities of the corresponding states in (a–d), respectively.

Previous studies show that the system Hamiltonian becomes defective at EPs whereat two or more eigenvalues and their corresponding eigenvectors coalesce into one [47]. The coalescence of states at the black star and yellow lines is reminiscent of the physics at the EPs and EAs in non-Hermitian systems. Looking into the details, Fig 2(a)–(d) plot Re[ω] for both the real (solid lines) and complex (dotted lines) solutions of (2) for different detuning and loss values. The solid lines in Fig. 2(a) for *l* = 0.7 show a typical bistable scenario. The red and blue solid lines correspond to stable and unstable states, respectively, based on computations of the Lyapunov exponents (see Section 1 within the online supplementary material). The red and blue solid lines coalesce at the two yellow points in Fig. 2(a) where only one state remains. We focus on the lower coalescence point (Δ_ω_ = 0.22) and plot Re[ω] as a function of loss in Fig. 2(b), wherein similarly two states coalesce into one while the higher red line remains. Meanwhile, we find that both Re[ω] and Im[ω] approach the corresponding coalescence point as $\sqrt{\epsilon }$, with ε being the perturbation on either the detuning or loss (see Section 2 within the online supplementary material), which thus indicates that the coalescence point is an order-2 EP (two eigenstates coalesce into one). As a straightforward extension, the two yellow lines in Fig. 1(b) should be arcs of EPs, i.e. EAs. These two EAs approach each other with increasing *l* and eventually merge at *l* = 1, where only one state remains at the coalescence point marked by the black star, as shown in Fig. 2(c) and (d). The critical scaling behavior can be obtained by analyzing (2), the secular equation, which itself is a cubic function of ω. This is reminiscent of the secular equation of systems possessing three resonators. At Δ_ω_ = 0, (2) is simplified as


(4) \begin{eqnarray*} \omega =\pm \sqrt{1-l^{2}},0; \end{eqnarray*}

and at *l* = 1, (2) reduces to


(5) \begin{eqnarray*} \omega ^{3}-\omega ^{2}\Delta _{\omega }-\Delta _{\omega }=0, \end{eqnarray*}

where compared to the third term, the second term can be ignored. Interestingly, Re[ω] and Im[ω] scale as $\sqrt[3] {\omega} $ and $\sqrt{l-1}$, respectively, when approaching Δ_ω_ = 0 and *l* = 1, indicating an anisotropic order-3 EP [48], which was originally believed to be possible only in three- or even higher-dimensional systems. In other words, we have realized higher-dimensional (≥3) exceptional physics within two coupled resonators with the aid of nonlinearity. Note that, though (1) and (2) have been discussed previously in the circumstance of wireless energy transfer [33] and optical bistability [34], the parameter regime containing such an order-3 EP and the corresponding physics have not been investigated previously.

To understand the underlying physics, we embed our two-resonance nonlinear system into a PT symmetric three-resonance linear Hamiltonian *H*_3*d*_. This can be done as *H*_3*d*_ shares the same manifold (eigenvalues and eigenvectors) as our two-resonator nonlinear system except for an auxiliary ‘neutral’ site as sketched in the lower panel of Fig. 1(a). Such a map can be derived as follows. (The proof is provided in Section 3 within the online supplementary material.) Let {ω_*i*_|(ψ_*i, A*_, ψ_*i, B*_)^*T*^}, where *i* = 1, 2, 3 and ‘^*T*^’ denotes the transpose, represent the three eigenvalues and eigenvectors of (1). Except for the parameters exhibiting EPs, there are always three solutions since (2) is cubic. The eigenvectors are not biorthogonal, and the space expanded by the eigenvectors is overcomplete. To construct the linear Hamiltonian *H*_3*d*_ and restore the biorthogonality, we extend the eigenstates by including an auxiliary ‘neutral’ site and the new eigenvectors are |φ_*i*_〉 = (α_*i*_ψ_*i, A*_, α_*i*_ψ_*i, B*_, ψ_*i, N*_)^*T*^, with $\alpha _{i}\in \mathbb {C}$ and ψ_*i, N*_ representing the wave amplitude inside the new site. The values of α_*i*_ and ψ_*i, N*_ can be uniquely determined by assuming that |φ_*i*_〉 satisfies the biorthogonal and normalized conditions. We denote by $|\phi _i^R\rangle$ the eigenvector of a three-resonance linear Hamiltonian $H_{3d}=\sum _{i=1}^{3}\omega _{i}|\phi _{i}^{R}\rangle \langle \phi _{i}^{L}|$, where $\langle \phi _i^L|$ is the left eigenvector of $|\phi _i^R\rangle$ following the biorthonormal condition. Such a Hamiltonian can be proven to be PT symmetric, and the PT operator is provided in Section 3 within the online supplementary material. We emphasize that *H*_3*d*_ defined above is unique for the corresponding nonlinear two-resonator system, and except for the EPs, such a construction works for any choice of loss and detuning.

With the map established, it is clear that three (two) eigenvalues and their corresponding eigenvectors coalesce at an order-3 (order-2) EP. Eigenstates at EPs are self-orthogonalized [15], and thus EPs can be identified with the vanishing of phase rigidity defined as$r_i=(\langle \phi _i^R |\phi _i^R\rangle )^{-1}$ [18,48–50]. Figure 2(e–h) show the phase rigidities along the loss and detuning direction, where we can see that the phase rigidities vanish at the EPs. A more detailed study shows that the critical exponents are 1/2 in Fig. 2(e–h) and 1/3 in Fig. 2(g) (Section 3 within the online supplementary material). Note that this order-3 EP exhibits different critical exponents of phase rigidity along the loss and detuning axes. Meanwhile, the EP line (yellow lines in Fig. 1(b)) consisting of normal order-2 EPs align well with the definition of EAs [50]. Hence, the order-3 EP is also the cusp singularity of multiple EAs that hence fits into the definition of EX [18].

### Enhanced signal-to-noise ratio

A HOEP, such as an EX, was proposed to magnificently enhance the sensor’s performance using the splitting of eigenfrequencies at the EP [21,35,36]. However, the implementation of EP-related sensing in various schemes faces challenges that have then triggered ongoing debates over the last few years. On the one hand, realizing higher-order EPs requires complex internal structures and tuning of a considerable number of parameters [5,21,35,43,44]. On the other hand, the precision of dispersion measurement is, in general, limited by the fundamental resolution limit [18,36,40,42,49] and other noise-induced bounds [41,45]. More specifically, the imaginary part of the eigenfrequencies will broaden the reflection or transmission spectrum [42]. Meanwhile, a nonvanishing imaginary part also indicates that the mode is decaying or amplifying, which limits the available measurement time, setting a fundamental resolution limit. Adding gain elements may overcome this experimental difficulty; however, they introduce additional noise, and then also lead to a degradation of the sensor due to the amplification of fluctuations and the coalescence of the eigenstates [37,39–41,51].

Here, we endeavor to address these challenges using the ‘EX’ point achieved with nonlinear saturable gain. Let us start with the parameter tuning issue. In our system, only the states represented by the solid red lines in Fig. 2(a–d) or, equivalently, the red surface in Fig. 1(b) are dynamically stable, i.e. can be experimentally probed. Any other excitations will fall back into one of the stable states within a short period considering that the gain is automatically adjusted with the wave amplitude. Nevertheless, the exceptional features such as Re[ω] scaling as $\sqrt[3] {\mathrm{\Delta }_\omega }$ associated with the ‘EX’ are still preserved for the stable states. Intriguingly, such an ‘EX’ point is reached with even fewer constraints or tuning parameters than for an order-2 EP, wherein, in addition to two tuning parameters, one needs additional efforts to enforce PT symmetry. In short, our ‘EX’ points do not require detail parameter tuning as other HOEPs in linear systems.

**Figure 3.**
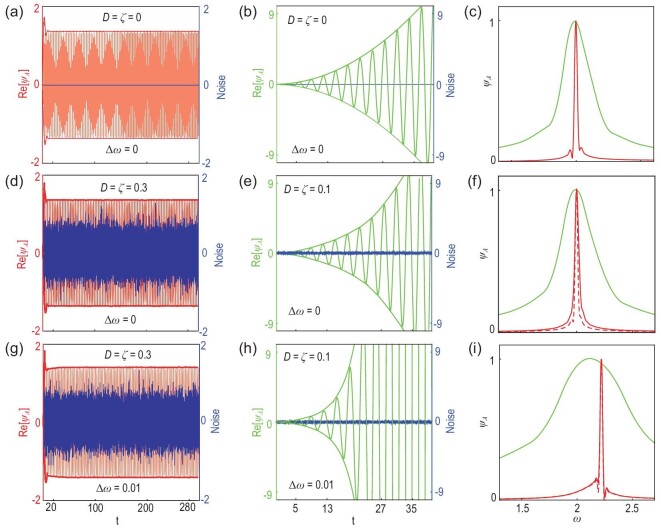
(a,d,g) The dynamics of Re[ψ_*A*_] (red lines) for the nonlinear Hamiltonian in (1) for different magnitudes of noise and detuning. (b,e,h) The dynamics of Re[ψ_*A*_] (green lines) for the corresponding linear Hamiltonian in Equation (S24) within the online supplementary material. The amplitudes of noise are given with the blue lines in the first two columns. (c,f,i) The corresponding Fourier spectra for the nonlinear (red) and linear (green) systems. Here the spectra are averaged over 100 independent noise realizations. The dynamics all begin with a small kick-start amplitude ψ_*A*_ = 10^−2^ and follow the corresponding linear or nonlinear Schrödinger equations thereafter. The Fourier spectra of the nonlinear systems (red lines) are obtained with the corresponding dynamics of Re[ψ_*A*_] within the period 0 ≤ *t* ≤ 300. For the linear systems, the wave magnitudes are increasing functions of time. We record the dynamics of Re[ψ_*A*_] until Max[|ψ_*A*_|^2^] = 100 is reached, and then perform the Fourier transform. In (a–f) Δ_ω_=0 and in (g–i) Δ_ω_ = 0.01. For (a–c), *D* = ζ = 0; for (d and g) and the solid red lines in (f and i), *D* = ζ = 0.3; and for (e and h), the solid green lines in (f and i), and the dashed red lines in (f and i), *D* = ζ = 0.1. For the nonlinear Hamiltonian, *l* = 1, ω_*B*_ = 2, and the gain saturation model is given by Equation (S6) within the online supplementary material.

Secondly, we analyze the spectrum broadening effect due to the imaginary parts of the eigenfrequencies. Figure 3(a) shows the dynamic of Re[ψ_*A*_] in our system with nonlinear saturable gain at zero detuning (Δ_ω_ = 0). The feedback mechanism of nonlinear saturable gain forces the system to reach and remain at one stable state that has vanishing imaginary part of the eigenfrequency. In contrast, the amplitude grows with time (∼*t*^2^) [37] at an EX of a linear system, as shown in Fig. 3(b). Here the EX of a linear system is realized within a coupled three-resonator system similarly as in [18]. The explicit form of the Hamiltonian is provided in Section 4 within the online supplementary material. Considering the fact that any detector has a finite measuring range, we set Max[|ψ_*A*_|^2^] = 100 to simulate the maximum energy that the detector can record. The exact value of the measuring range does not change the overall conclusion. The corresponding spectrum of the linear system is shown in Fig. 3(c) with the green line, where an obvious broadening is observed. In contrast, since only one stable state is reached in our nonlinear system, we can obtain the desired resolution for an arbitrarily long time (see the red line in Fig. 3(c)). Another annoying issue in practice for both the linear and nonlinear systems is the back action of the readout component that may possibly change the gain model. Interestingly, the eigenfrequency ω of the steady state in our nonlinear system is irrelevant for the specific form of saturated gain provided that (3) is satisfied. The system will eventually reach and remain in one stable state independent of whether the readout component is connected or not. This merit allows us to conduct dispersion measurement without any shift of the eigenfrequencies.

Lastly, let us proceed to see the effect of noise. The unavoidable fluctuations, i.e. noise, will also degrade the precision, especially in the vicinity of an EX and when gain elements are involved. In the context of optics and photonics, sensing is typically viewed as a scattering process of the input photon, and the linewidth of the beating frequency due to noise is increased by the Petermann factor (PF) and thereby reduces the SNR [38]. In our nonlinear non-Hermitian Hamiltonian, the PF of the stable state is shown to be PF ∝ |Δ_ω_|^−2/3^ near the EX, which diverges (see Section 4 within the online supplementary material). However, the divergence of PF cannot overshadow the enhanced SNR. More specifically, the signal-enhancement factor (SEF) [38] is given by |∂ω/∂Δ_ω_|^2^ ∝ |Δ_ω_|^−4/3^, which is a higher-order divergence compared to PF. Hence, (SNR)^−1^, defined as (PF/SEF)^1/2^ is proportional to |Δ_ω_|^1/3^ when approaching the ‘EX’. In other words, not only the responsibility, i.e. SEF, but also the SNR is dramatically improved when approaching the nonlinear ‘EX’ along the Δ_ω_ direction. The purple line in Fig. 1(c) shows (SNR)^−1^ versus Δ_ω_ wherein we can see that the purple line vanishes at the ‘EX’. Note that the PF of the stable state diverges at Δ_ω_ = 0, and thus one would expect that the fluctuation introduced by the noise is infinite. In the presence of noise, the gain coefficient shifts away from the EX condition and then the PF decreases rapidly (the corresponding states are no longer self-orthogonalized). As a result, the amplitude of noise at Δ_ω_ = 0 actually only contributes to a finite line width. In short, the minimum detectable signal should be even smaller than the red line in Fig. 1(c) in practice. In contrast, (SNR)^−1^ reaches a finite value for the EX in the linear system (green dashed line in Fig. 1(c)) since PF and SEF exhibit the same critical component (∝|Δ_ω_|^−4/3^) when approaching the linear EX. Note that such guidance discussed above is commonly used in quantum metrology [52–54], but is not the only measurement scheme in classical wave systems [55].

In our case, we can evaluate the efficacy of sensors from the system dynamics. Here the SNR is defined as


(6) \begin{eqnarray*} (\text{SNR})^{-1}=\frac{\delta \varepsilon _{\omega }}{|\partial \omega /\partial \Delta _{\omega }|}, \end{eqnarray*}

where standard deviation $\delta \varepsilon _\omega = \text{FWHM}/ (2\sqrt{2ln2})$ with FWHM and ω representing the line width and the center frequency for the spectrum obtained from the Fourier transform of the time-dependent wave function, respectively. Figure 3(d–i) show the dynamics of Re[ψ_*A*_] for both the nonlinear (red) and linear (green) systems, and the corresponding Fourier spectra (average over 100 independent noise realizations) in the presence of noise. We also provide the corresponding noise function with the blue lines for reference. Here, we exaggerate the noise amplitude in nonlinear simulations for demonstration purposes. Even if the noise amplitude is almost half of that of the stable state at *D* = ζ = 0.3, the effect of noise is still largely suppressed, as can be seen from the envelope of Re[ψ_*A*_]. This is due to the fact that any derivation from the stable states as introduced by the noise is unstable (see the Lyapunov exponents analysis in Section 1 within the online supplementary material). At Δ_ω_ = 0.01 away from the EX, the effect of noise is significantly suppressed by the feedback mechanism, wherein we can see that the spectra for *D* = ζ = 0.3 (solid red line) and *D* = ζ = 0.1 (dashed red line) almost overlap with each other in Fig. 3(i). For a more systematic study, we perform simulations for different Δ_ω_ at *D* = ζ = 0.3 for 100 independent noise realizations, and then obtain the averaged center frequency and line width. (See Section 4 within the online supplementary material.) With (6), we retrieve the SNR from the system dynamics, as shown with the purple asterisks in Fig. 1(d). Here the fluctuation of SNR is introduced by the finite simulation time. (See Section 4 within the online supplementary material.) With a longer simulation time, the SNR will be further improved. In a short summary, the actual SNRs retrieved from the system dynamics are dramatically improved when approaching the nonlinear ‘EX’ along the Δ_ω_ direction.

### Experimental demonstrations

**Figure 4.**
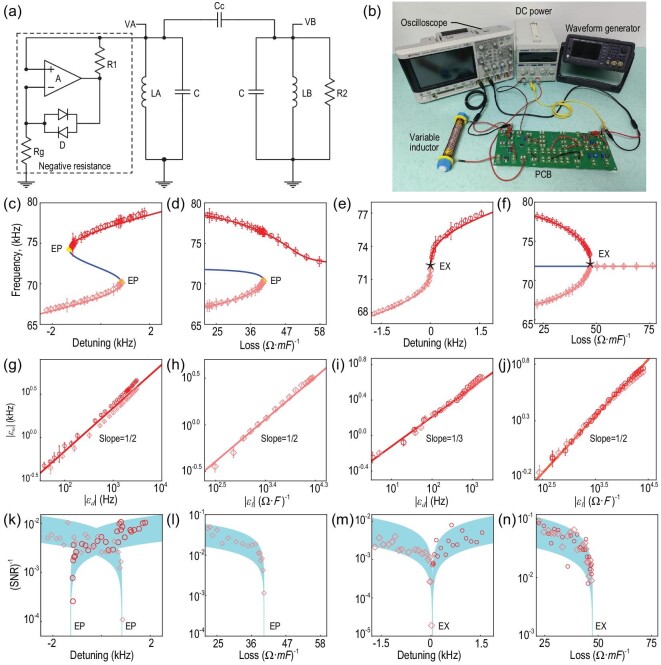
(a) Circuit used for experimental verification, showing the inductors (L), capacitors (C), resistors (R), diodes (D) and amplifier (A). (b) Photo of the experimental setup. (c–f) Measured resonance frequencies (open circles and diamonds) of the system together with the steady-state eigenfrequencies from the simulations (solid lines). Loss = 35.7 (Ω · mF)^−1^ in (c), detuning = 0.36 (kHz) in (d), loss = 47.5 (Ω · mF)^−1^ in (e) and detuning = 0 (kHz) in (f). (g–j) The critical behavior near the corresponding EPs and EX in (c–f), respectively. The solid lines are the theoretical predictions. The estimated experimental errors (standard deviation obtained from eight independent measurements) in (c–j) are smaller than the marker sizes. For demonstration purposes, we exaggerate the error bars by factors of 100, 100, 50, 50, 10, 10, 10 and 10 in (c–j), respectively. (k–n) The corresponding SNR-1 for different detuning and loss values. The cyan background represents the theoretical fitting as shown similarly in Fig. 1(d).

The above-discussed physics is general, and here we realize it with a circuit system, as shown in Fig. 4(a). The system consists of two LC resonators coupled by a capacitor *C_c_*. The LC resonator on the right-hand side is lossy, while the resonator on the left-hand side exhibits saturable gain realized through an effective negative resistor (Section 5 within the online supplementary material). Voltages *V_A_* and *V_B_* represent the wave amplitudes inside the left and right resonators, respectively. The Kirchhoff equations that describe the dynamics of such a circuit can be mapped into the coupled mode equation, i.e. (1), by assuming that *C_c_* ≪ *C*. Under this mapping, the resonance frequency, coupling, loss and gain are given by $\omega _{A,B}=1/\sqrt{L_{A,B}C}$, κ = ω_*B*_*C_c_*/2*C, l* = 1/2*R_B_C* and *g*[*V_A_*] = *R_D_*[*V_A_*]/2*R_g_R*_1_*C*, respectively (Section 6 within the online supplementary material). The gain *g*[*V_A_*] decreases with increasing *V_A_*, as *R_D_*[*V_A_*] is a monotonic decreasing function of *V_A_* over the range of interest (Section 5 within the online supplementary material). Figure 4(b) shows the experimental setup, where the oscilloscope measures the resonance frequency and the amplitudes of *V_A_* and *V_B_*, the DC power supplies power for the amplifier and the waveform generator is used to excite the selected mode. We also add a homemade variable inductor for fine tuning of *L_A_* and variable resistors to control the loss and detuning. Details of the circuit elements on the PCB can be found in Section 7 within the online supplementary material.

Figure 4(c–f) show the measured resonance frequencies for various detuning and loss values for the corresponding cases studied in Fig. 2(a–d), respectively. The open circles and diamonds are measured resonance frequencies, and the red and blue solid lines represent the stable and unstable steady states of the corresponding Kirchhoff equations, respectively. The unstable steady states cannot be reached experimentally, and the two bistable states can be selectively excited by a ‘kicking’ process with an external waveform generator (Section 8 within the online supplementary material). Figure 4(g–j) show the critical behavior near the ‘EAs’ and ‘EX’. The slopes are fit to 1/2 for points on ‘EAs’ along both the detuning and loss axes, and the slopes for the ‘EX’ are 1/3 along the detuning axes and 1/2 along the loss axes that verifies the eigenvalue anisotropy of the ‘EX’. Meanwhile, we also measured the ratio of the voltages on both resonators, which also agrees almost perfectly with the simulations (Section 9 within the online supplementary material). Consistent with the simulations of linewidth (Fig. S6 within the online supplementary material), the noise effect can be compensated by the feedback mechanism of the saturable gain. The experimental errors (standard deviation obtained from eight independent measurements) are smaller than the marker sizes. For demonstration purposes, we exaggerate the error bars by factors of 100, 100, 50, 50, 10, 10, 10 and 10 in Fig. 4(c–j), respectively. Figure 4(k–n) show the corresponding SNR-1 for different detuning and loss values, which once again prove that the feedback mechanism can give a solution to the ongoing debate of linear EPs on the impact of noise. Although only the circuit system is observed, our result can be generalized to diverse classical systems, ranging from photonics [56], mechanics [57] and acoustics [58] to active matter [59]. And, especially in optics, with the rapid development of metastable lasers [60,61] and nonlinear resonators [62], the possibility is even more conceivable.

## DISCUSSION

In summary, we show with theory and experiments that a nonlinear ‘EX’ with ultra-enhanced SNR can be realized within a two-resonance non-Hermitian system by incorporating nonlinear saturable gain. The nonlinear gain introduces bistable steady states and another unstable state that effectively extends the dimension of the system and enables the investigation of HOEPs. Our work shows that the unstable steady states, which were generally considered to be irrelevant to the dynamics, have definite contributions to the exceptional features of non-Hermitian systems. The possibility of exploring even more fascinating ‘exceptional’ constituents in simple nonlinear non-Hermitian systems is thus conceivable. Highlighting the fundamental understanding of nonlinear non-Hermitian systems, exceptional singularities and dimension, our findings also constitute a significant advance to realizing HOEP sensors with magnificently enhanced SNR.

## Supplementary Material

nwac259_Supplemental_File
