# Supplementary material for: Nonlinearity-enabled higher-order exceptional singularities with ultra-enhanced signal-to-noise ratio
Source: Natl Sci Rev. 2022 Nov 16;10(7):nwac259. doi: 10.1093/nsr/nwac259 (PMC10232044; doi:10.1093/nsr/nwac259)
Supplement: nwac259_Supplemental_File [file nwac259_supplemental_file.docx]

**Supplementary Information**

**“****Nonlinearity enabled ultrasensitive higher-order exceptional singularities”**

Kai Bai,^1^ Liang Fang,^1^ Tian-Rui Liu,^1^ Jia-Zheng Li,^1^ Duanduan Wan,^1*^ Meng Xiao,^1, 2†^

*^1^Key Laboratory of Artificial Micro- and Nano-structures of Ministry of Education and School of Physics and Technology, Wuhan University, Wuhan 430072, China*

*^2^Wuhan Institute of Quantum Technology, Wuhan 430206, China*

**This Supplementary Information contains 10 sections, 17 figures, and 13 references.**

**CONTENTS**

1. **Stability analysis.**
2. **Theoretical analysis of the exceptional arcs and exceptional nexus.**
3. **The map between a two-resonator nonlinear system and a three-resonator linear system.**
4. **Petermann-factor of stable states and Linear PT Hamiltonian with an EX.**
5. **Negative resistance.**
6. **Derivation of the coupled-mode equations.**
7. **Circuit elements on the PCB.**
8. **Switching between stable states.**
9. **Measured eigenfrequencies versus circuit parameters.**
10. **Experimental error analysis.**
11. **Stability** **analysis.**

In this section, we study the stabilities of steady states from the system dynamics of tight binding model using the Lyapunov exponent[1,2]. The system dynamics follow the time-dependent nonlinear Schrödinger equations,

$$\begin{aligned} \frac{d}{dt}\left( \begin{matrix} \psi_{A} \\ \psi_{B} \end{matrix} \right)=-i\left( \begin{matrix} \omega_{A}+ig\left( \left| \psi_{A} \right| \right) & \kappa\\ \kappa& \omega_{B}-il \end{matrix} \right)\left( \begin{matrix} \psi_{A} \\ \psi_{B} \end{matrix} \right),\#\left( S1 \right) \end{aligned}$$

where $g\left( \left| \psi_{A} \right| \right)$ represents the amplitude dependent gain. To analyze the stability of a steady-state solution $\tilde{\psi}_{A,B}$ at frequency $\omega$, we add a time-dependent perturbation on the steady state as

$$\begin{aligned} \psi_{A,B}=\tilde{\psi}_{A,B}+\rho_{A,B}\left( t \right).\#\left( S2 \right) \end{aligned}$$

Insert Eq. (S$2$) into Eq. (S$1$), omit terms that are quadratic or higher-order in $\rho_{A,B}\left( t \right)$, and in a frame rotating at $\omega$, we end up with a pair of time-dependent equations,

$$\begin{aligned} \frac{d}{dt}\rho_{A}=A\rho_{A}\left( t \right)+B\rho_{A}^{*}\left( t \right)+C\rho_{B}\left( t \right), \\ \frac{d}{dt}\rho_{B}=C\rho_{A}\left( t \right)+D\rho_{B}\left( t \right),\#\left( S3 \right) \end{aligned}$$

where

$$\begin{aligned} A=-i\left( \omega_{A}-\omega\right)+g\left[ \left| \tilde{\psi}_{A} \right| \right]+\frac{1}{2}\left. \frac{dg}{d\left| \psi_{A} \right|} \right|_{\tilde{\psi}_{A}}\left| \tilde{\psi}_{A} \right|, \\ B=\left. \frac{dg}{d\left| \psi_{A} \right|} \right|_{\tilde{\psi}_{A}}\frac{\tilde{\psi}_{A}^{2}}{2\left| \tilde{\psi}_{A} \right|}, \\ C=-i\kappa, \\ D=-i\left( \omega_{B}-\omega\right)-l. \#\left( S4 \right) \end{aligned}$$

We assume that the perturbation has exponential time-dependence $e^{\lambda t}$, i.e., $\rho_{A,B}\left( t \right)=\tilde{\rho}_{A,B}e^{\lambda t}$ where $\lambda$ is referred to as the Lyapunov exponent. Noting that $\tilde{\rho}_{A,B}$ are complex, $\lambda$ satisfies

$$\begin{aligned} \left( \begin{matrix} A & B & \begin{matrix} C & 0 \end{matrix} \\ B^{*} & A^{*} & \begin{matrix} 0 & C^{*} \end{matrix} \\ \begin{matrix} C \\ 0 \end{matrix} & \begin{matrix} 0 \\ C^{*} \end{matrix} & \begin{matrix} \begin{matrix} D \\ 0 \end{matrix} & \begin{matrix} 0 \\ D^{*} \end{matrix} \end{matrix} \end{matrix} \right)\left( \begin{matrix} \tilde{\rho}_{A} \\ \tilde{\rho}_{A}^{*} \\ \begin{matrix} \tilde{\rho}_{B} \\ \tilde{\rho}_{B}^{*} \end{matrix} \end{matrix} \right)=\lambda\left( \begin{matrix} \tilde{\rho}_{A} \\ \tilde{\rho}_{A}^{*} \\ \begin{matrix} \tilde{\rho}_{B} \\ \tilde{\rho}_{B}^{*} \end{matrix} \end{matrix} \right).\#\left( S5 \right) \end{aligned}$$

For a stable steady state, all the perturbations are decaying, i.e., $Re(\lambda) < 0$ except for one with $\lambda$ = 0, which arises from the undetermined global phase[1].

In the following, we calculate the real parts of Lyapunov exponents for two models explicitly to demonstrate the stabilities of steady states. The first model we considered is the commonly used gain saturation model[3] where the amplitude dependent gain function is given by

$$\begin{aligned} g\left[ |\psi_{A}| \right]=\frac{2\left( g_{1}+g_{10} \right)}{1+\left| \psi_{A} \right|^{2}}-g_{10}.\#\left( S6 \right) \end{aligned}$$

where $g_{10}$ represents the intrinsic loss rate and $g_{1}$ represents the overall gain rate at $\left| \psi_{A} \right|=1$. In the second model, we extract the amplitude dependent gain function from our experimental setup where the gain function can be approximated as

$$\begin{aligned} g\left[ \left| \psi_{A} \right| \right]=\frac{a}{1+b\left| \psi_{A} \right|}-c.\#\left( S7 \right) \end{aligned}$$

Here *c* represents the intrinsic loss rate, *a* and *b* are real fitting parameters. Here, for simplicity, the model is normalized by the coupling rate $\kappa$ and thus {*a*, *b*, *c*} are dimensionless numbers. Figure S1 shows the fitting of the gain value versus $\left| \psi_{A} \right|$ (open circles) with the gain model (solid line) in Eq. (S$7$). We can see that such a simple model works pretty well inside the range of interest. The details of how we get the gain value from simulations are provided in Sec. 3 below.


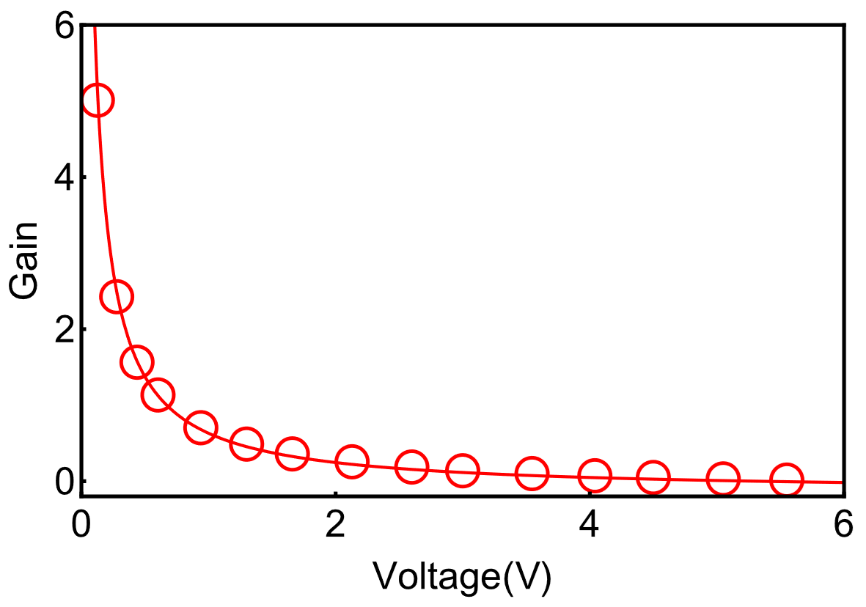


**Figure S1.** The open circles are data from the simulations using the circuit elements in our experiment. The solid line is the fitting curve using Eq. (S$7$) with *a*=32.4, *b*=40, and *c*=0.15.

Instead of $\left| \psi_{A} \right|^{2}$ dependence of gain in Eq. (S$6$), here, the lowest order of $\left| \psi_{A} \right|$ is linear in Eq. (S$7$) for the circuit we implemented. On the other hand, both gain models are monotonic decreasing functions of the wave amplitude and the stabilities of the steady states actually do not depend on the specific gain model we use. Moreover, since the frequencies of the steady states are obtained solely by solving Eq. (2), the frequencies are also independent of the specific gain model as long as Eq. (3) can be satisfied by adjusting the wave amplitude. Figure S2 shows the calculated Lyapunov exponents for the above two gain models, where the first row shows the frequencies of steady states versus detuning or loss for the four typical cases considered in the main text, and the second and third rows show the real parts of Lyapunov exponents of gain models in Eqs. (S$6$) and (S$7$), respectively. Here, we use different colors to label each state, red and light red for stable steady states and blue for unstable steady states. Each state has four Lyapunov exponents with one being zero not shown here, and we can see that at least one of the blue curves for each case in the second and third rows are above zero and all the red and light red curves are below zero. Thus, we can conclude that the states marked by the blue curves are unstable and the red and light red curves stable.


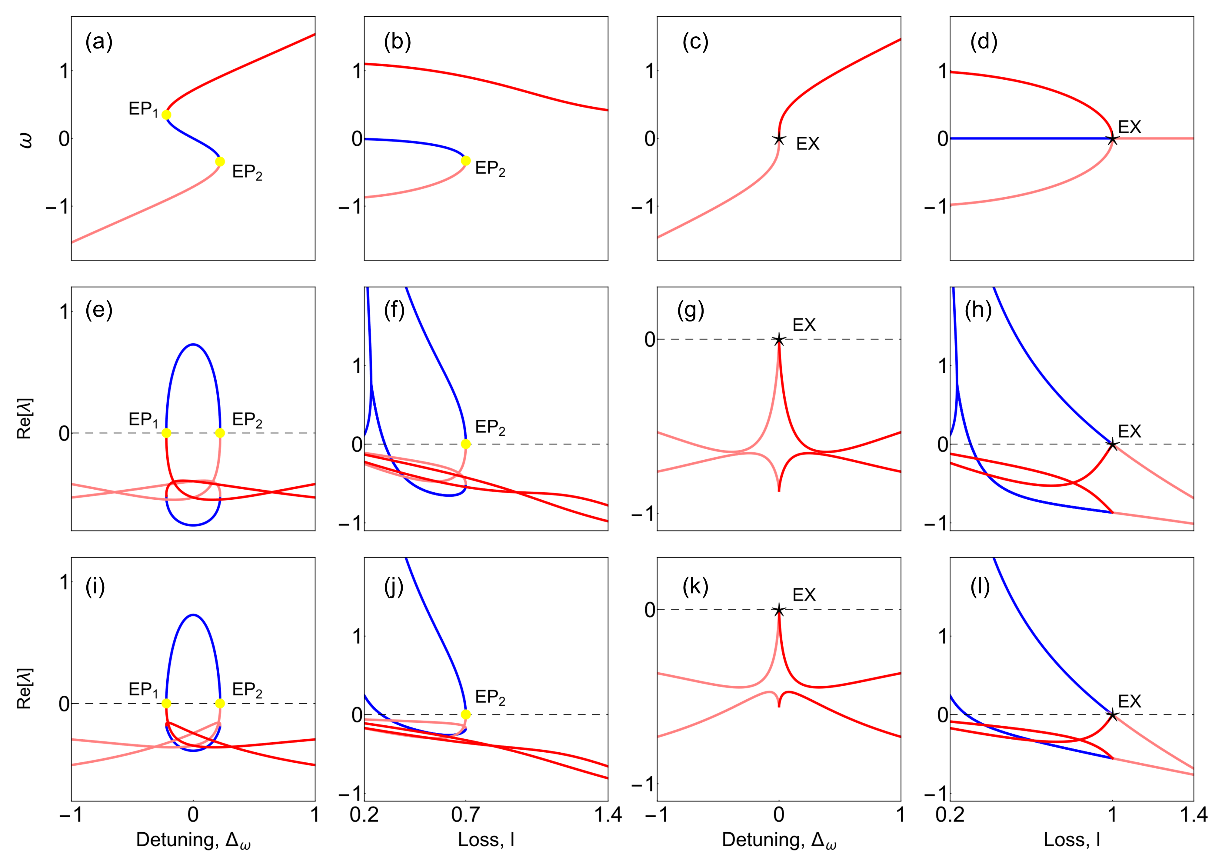


**Figure S2.** (a-d) The frequency of steady states ω versus the detuning and loss. The (light) red and blue solid lines represent stable and unstable steady states, respectively. The corresponding Lyapunov exponents are shown in (e-h) for the gain saturation model in Eq. (S$6$) and in (i-l) for the gain saturation model in Eq. (S$7$). Each state has three Lyapunov exponents and two of which have degenerated $\mathrm{Re}\left( \lambda\right)$. At least one of the Lyapunov exponents satisfy $\mathrm{Re}\left( \lambda\right)> 0$ for unstable states (blue lines), and all the Lyapunov exponents satisfy $\mathrm{Re}\left( \lambda\right)< 0$ for stable states (red and light red lines). At the EPs and EX point, the eigenmodes coalesce, resulting in marginal stability along with frequency bifurcation. $l=0.7$ in (a, e, i), $\Delta_{\omega}=0.22$ in (b, f, j), $l=1$ in (c, g, k), $\Delta_{\omega}=0$ in (d, h, l). $g_{1}=3$ and $g_{10}=0.05$ in (e-h), and $a=32.4$, $b=40$ and $c=0.15$ in (i-l).

1. **Theoretical analysis of the exceptional arcs and exceptional nexus.**

In this section, we analyze the trajectories of exceptional arcs (EAs) and the existence of exceptional nexus (EX). We start with the nonlinear Schrödinger equation with gain depending on the amplitude of resonator A:

$$\begin{aligned} \left( \begin{matrix} \omega_{A}+ig\left[ \left| \psi_{A} \right| \right] & \kappa\\ \kappa& \omega_{B}-il \end{matrix} \right)\left( \begin{matrix} \psi_{A} \\ \psi_{B} \end{matrix} \right)=\omega\left( \begin{matrix} \psi_{A} \\ \psi_{B} \end{matrix} \right).\#\left( S8 \right) \end{aligned}$$

The eigenfrequency $\omega$ satisfies

$$\begin{aligned} \left( \omega-\omega_{A} \right)\left( \omega-\omega_{B} \right)^{2}+\left( \omega-\omega_{A} \right)l^{2}-\kappa^{2}\left( \omega-\omega_{B} \right)=0.\#\left( S9 \right) \end{aligned}$$

Solving this equation, we assume that the gain is adjustable by changing the wave amplitude $\left| \psi_{A} \right|$ such that Eq. (S$9$) is satisfied at $g=g_{s}= l(\omega_{A}-\omega)/(\omega_{B}-\omega)$. For convenience, we set$\omega_{B}$=0, and $\omega_{A}$ and $l$ are normalized by the parameter $\kappa$. We also define $\delta_{l}=l-1$ and $\Delta_{\omega}=\omega_{A}-\omega_{B}=\omega_{A}$, and Eq. (S$9$) becomes

$$\begin{aligned} \omega^{3}-\Delta_{\omega}\omega^{2}+(2\delta_{l}+\delta_{l}^{2})\omega-\left( 1+\delta_{l} \right)^{2}\Delta_{\omega}=0.\#\left( S10 \right) \end{aligned}$$

We adapt the Sylvester matrix^4^ to identify the locations of exceptional points (EPs). The right hand of Eq. (S$10$) is defined as the characteristic polynomial

$$\begin{aligned} p\left( \omega\right)=a_{3}\omega^{3}+a_{2}\omega^{2}+a_{1}\omega+a_{0},\#\left( S11 \right) \end{aligned}$$

where $a_{3}=1$, $a_{2}=-\Delta_{\omega}$, $a_{1}=2\delta_{l}+\delta_{l}^{2}$ and $a_{0}=-\left( 1+\delta_{l} \right)^{2}\Delta_{\omega}$. The derivative of $p\left( \omega\right)$ is thus

$$\begin{aligned} q\left( \omega\right)=b_{2}\omega^{2}+b_{1}\omega+b_{0},\#\left( S12 \right) \end{aligned}$$

where $b_{2}=3$, $b_{1}=2a_{2}$ and $b_{0}=a_{1}$. The locations of all EPs can be determined by requiring the discriminant of the characteristic polynomial $p$, $-\det\left[ \mathrm{Syl}\left( p,q \right) \right]$, to vanish. Here, $\mathrm{Syl}\left( p,q \right)$ is short for the Sylvester matrix of the polynomials $p$ and $q$, which has the following form

$$\begin{aligned} \mathrm{Syl}\left( p,q \right)=\left( \begin{matrix} a_{3} & a_{2} & \begin{matrix} a_{1} & a_{0} & 0 \end{matrix} \\ 0 & a_{3} & \begin{matrix} a_{2} & a_{1} & a_{0} \end{matrix} \\ \begin{matrix} b_{2} \\ 0 \\ 0 \end{matrix} & \begin{matrix} b_{1} \\ b_{2} \\ 0 \end{matrix} & \begin{matrix} \begin{matrix} b_{0} \\ b_{1} \\ b_{2} \end{matrix} & \begin{matrix} 0 \\ b_{0} \\ b_{1} \end{matrix} & \begin{matrix} 0 \\ 0 \\ b_{0} \end{matrix} \end{matrix} \end{matrix} \right).\#\left( S13 \right) \end{aligned}$$

Explicitly, the locations of all the EPs should satisfy

$$\begin{aligned} -\det\left[ \mathrm{Syl}\left( p,q \right) \right]=-32{\delta_{l}}^{3}-48{\delta_{l}}^{4}-24{\delta_{l}}^{5}-4{\delta_{l}}^{6}-27{\Delta_{\omega}}^{2}-4{\Delta_{\omega}}^{4}-72\delta l{\Delta_{\omega}}^{2} \\ -68{\delta_{l}}^{2}{\Delta_{\omega}}^{2}-32{\delta_{l}}^{3}{\Delta_{\omega}}^{2}-8{\delta_{l}}^{4}{\Delta_{\omega}}^{2}-8\delta_{l}{\Delta_{\omega}}^{4}-4{\delta_{l}}^{2}{\Delta_{\omega}}^{4}=0,\#\left( S14 \right) \end{aligned}$$

Note that here, the discriminant is real instead of complex, which thus reduces the conditions of forming an order-2 EP from two equations to only one equation. Meanwhile, we can observe that the shape of the EAs (if exist) should be symmetric about $\Delta_{\omega}=0$ since $\det\left[ \mathrm{Syl}\left( p,q \right) \right]$ has only even order of $\Delta_{\omega}$. However, such the symmetry for the trajectories of EAs cannot be extended to the spectra or states since these two EAs involve the coalescence of different states (see Fig. 1(b) in the main text). To further our study, let us consider a few special cases.

*Case I*, near $\left( \delta_{l}, \Delta_{\omega} \right)=\left( 0,0 \right)$. Keeping only the lowest orders of $\delta_{l}$ and $\Delta_{\omega}$ for Eq. (S$14$), we have

$$\begin{aligned} 32\left( \delta_{l} \right)^{3}+27\left( \Delta_{\omega} \right)^{2}\approx0.\#\left( S15 \right) \end{aligned}$$

Thus, one can conclude that the two EAs $\Delta_{\omega}\approx\pm\sqrt{-{32\delta}_{l}^{3}/27}$ form an ordinary cusp at $\left( \delta_{l}, \Delta_{\omega} \right) =\left( 0,0 \right)$. At such a point, two out of three states become defective which hence corresponds to an order-3 EP (EP3).

*Case II*, at $\Delta_{\omega}=0$. Equation (S$10$) reduces to

$$\begin{aligned} \omega=\pm\sqrt{-\left( 2\delta_{l}+\delta_{l}^{2} \right)}, 0.\#\left( S16 \right) \end{aligned}$$

Hence Eq. (S$10$) has three real solutions when $\delta_{l}<0$, and two of them become complex when $\delta_{l}>0$. Except for the solution $\omega=0$, both the real and complex parts of the other two solutions vary as $\left( \delta_{l} \right)^{1/2}$ when approaching the EP3.

*Case III*, at $\delta_{l}=0$. Equation (S$10$) reduces to

$$\begin{aligned} \omega^{3}-\omega^{2}\Delta_{\omega}-\Delta_{\omega}=0.\#\left( S17 \right) \end{aligned}$$

Near the EP3 where $\omega=0$, the second term can be ignored, and the solutions vary as $\left( \Delta_{\omega} \right)^{1/3}$ when approaching the EP3.

*Case IV*, at the EAs. Two of the roots of Eq. (S$10$) collapse at $\omega_{EAs}$ and the other root remains at $\omega_{3}$ (not at exactly the EX). Thus Eq. (S$10$) can be rewritten as

$$\begin{aligned} f\left( \Delta_{\omega,EAs},\delta_{l,EAs},\omega_{EAs} \right)=\left( \omega-\omega_{EAs} \right)^{2}(\omega-\omega_{3})=0,\#\left( S18 \right) \end{aligned}$$

In the vicinity of EAs, we have

$$\begin{matrix} f\left( \Delta_{\omega,EAs}+\Delta_{\omega,shift},\delta_{l,EAs}+\delta_{l,shift},\omega_{EAs}+\omega_{shift} \right)=f\left( \Delta_{\omega,EAs},\delta_{l,EAs},\omega_{EAs} \right) \\ +\left. \frac{\partial f}{\partial\Delta_{\omega}} \right|_{\Delta_{\omega,EAs}}\Delta_{\omega,shift}+\left. \frac{\partial f}{\partial\delta_{l}} \right|_{\delta_{l,EAs}}\delta_{l,shift}+\left. \frac{\partial^{2}f}{\partial\omega^{2}} \right|_{\omega_{EAs}}\omega_{shift}^{2}+O\left( \Delta_{\omega,shift}, \delta_{l,shift},\omega_{shift}^{2} \right)=0 \end{matrix}, \left( S19 \right)$$

where $O(\cdot)$ are higher-order terms, and $\left. \frac{\partial f}{\partial\omega} \right|_{\omega_{EAs}}=0$ since $q\left( \omega_{EAs} \right)=0$ according to Eq. (S$12$). Thus,$\omega_{shift}$ shows a square-root dependence along both the $\delta_{l}$ direction (at $\Delta_{\omega,shift}$=0) and the $\Delta_{\omega}$ direction (at $\delta_{l,shift}=0)$.

Observing that the coefficients of Eq. (S$10$) are all real, the complex solutions of Eq. (3) (if any) always come in complex conjugate pairs. Figure S3(a-d) and (e-f) show the real and imaginary parts of the solutions of Eq. (S$10$) as functions of detuning and loss, respectively. We can see clearly that one of the solutions is always real while the other two can be either both real or form a complex conjugate pair. Figure S3(i-l) show the critical behaviors around the EAs and EP3, which are 1/2 along both the detuning and loss axis for EPs on the EAs, and 1/3 along the detuning axis and 1/2 along the loss axis for the EP3 consistent with Eq. (S$16$) and (S$17$).


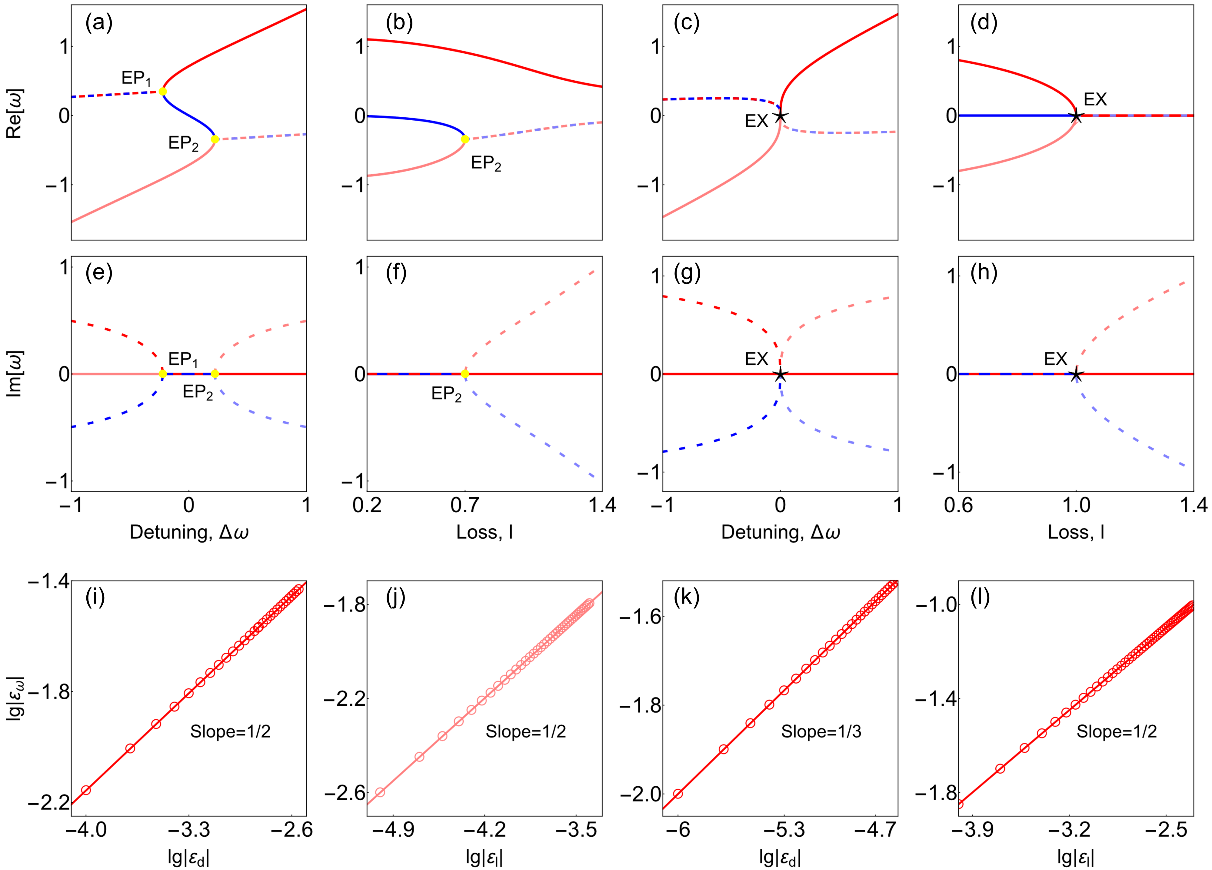


**Figure S3.** Real part (a-d) and imaginary part (e-f) of the solutions of Eq. (S$9$) versus the detuning and loss. (i-l), The critical behavior near the corresponding EPs and EP3 can be characterized by the slope on the logarithmic scale. The red and blue solid lines represent the stable and unstable steady states, respectively, and red and blue dashed lines represent complex solutions of Eq. (S$9$) with positive and negative imaginary parts, respectively. $l=0.7$ in (a, e, i), $\Delta_{\omega}=0.22$ in (b, f, j), $l=1$ in (c, g, k), $\Delta_{\omega}=0$ in (d, h, l), and all the other parameters are the same as in Fig. 1(b).

1. **The map** **between a two-resonator nonlinear system and a three-resonator linear system.**

In this section, we illustrate the map between a two-resonator nonlinear system and a three-resonator linear system. First, we start with a typical three-resonator linear system, as shown in Fig. S4(a). The eigenvalues {$\omega$i } and eigenvectors {(·)i}, and the exceptional topological constituents (such as the EPs, EAs, and EX) of such a system have been investigated extensively [4–9]. Now imagine that the field amplitude inside resonator C (the leftmost one) cannot be measured as sketched in Fig. S4(b). However, if we can identify the eigenvectors and measure the fields inside resonators A and B, the amplitude in resonator C for each eigenvector can be determined by biorthonormal condition. Note that the phase of the field in resonator C is irrelevant as it can be canceled by a global phase factor of the eigenvector. In other words, no information is missing if one only cares about the topology of a system which is associated entirely with the eigenspace, i.e., eigenvalues and eigenvectors. Then we go one step further and assume that we have a nonlinear system in Fig. S4(c) which shares the same $\left\{ \omega_{i}|\left( \psi_{i,A},\psi_{i,B} \right) \right\}$ as the linear system in Fig. S4(b). Thus in all, we have a one-to-one mapping between three-resonator linear system in Fig. S4(a) and a two-resonator nonlinear system in Fig. S4(c). Note here though the two-resonator nonlinear system only covers a subspace of the three-resonator linear system, the HIT associated with the EX can already be verified inside such a subspace.


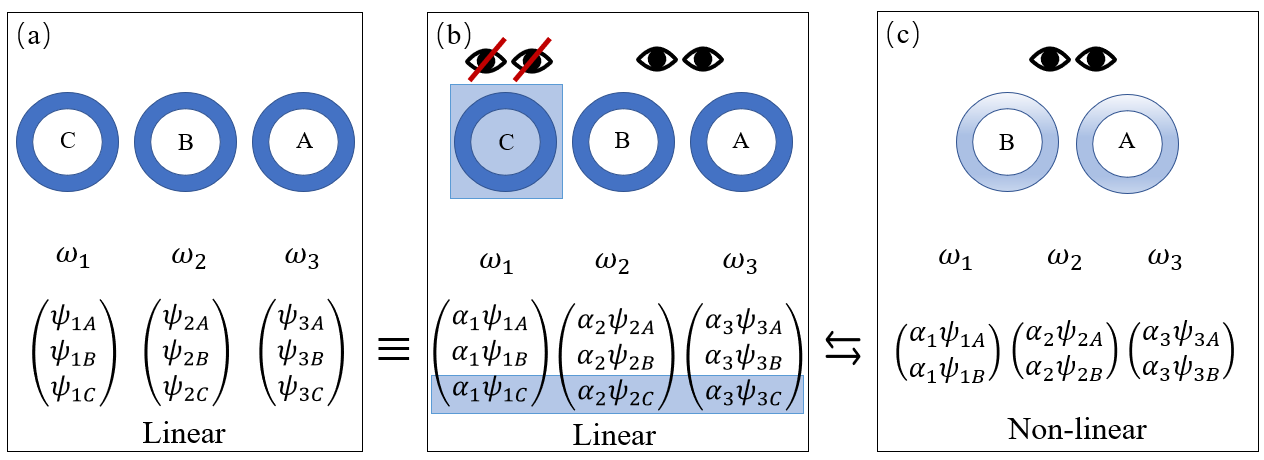


**Figure S4.** (a), A three-resonator (labeled by A B, and C, respectively) linear system with eigenspace {$\omega$*_i_*} and {(·)*_i_*}denoting the eigenvalues and eigenvectors, respectively. (b), Since the amplitude of $\{\psi_{i,C}\}$ can be obtained from the biorthonormal condition, no information of the eigenspace is missing if only the fields in resonators A and B can be probed. (c), One can have a nonlinear two-resonator system which shares the same $\left\{ \omega_{i}|\left( \psi_{i,A},\psi_{i,B} \right) \right\}$ as the three-resonator system in (b).

Following the idea discussed above, now we start with the two-resonator nonlinear system. For any loss and detuning not on the EAs and EP3, we construct three new vectors $\left| \phi_{i} \right\rangle=\left( \alpha_{i}\psi_{i,A},\alpha_{i}\psi_{i,B},\psi_{i,N} \right)^{\text{T}}$ with $\alpha_{i}\in\mathbb{R}$ and $\psi_{i,N}$ representing the wave amplitude inside a new site. These three vectors satisfy both the biorthogonal and normalized conditions, which in total have nine independent equations (due to the symmetry), and thus $\alpha_{i}$ and $\psi_{i,N}$ can be uniquely determined. The three-resonance linear Hamiltonian as

$$\begin{aligned} H_{3d}=\sum_{i=1}^{3} \omega_{i}|\phi_{i}^{R}\rangle\langle\phi_{i}^{L}|,\#\left( S20 \right) \end{aligned}$$

where $\langle\phi_{i}^{L}|$ is the left eigenvector of $\left| \phi_{i}^{R} \right\rangle$ following the orthonormal condition. The characteristic polynomial of $H_{3d}$ are the same as $p\left( \omega\right)$ in Eq. (S$11$) since they share the same eigenfrequencies. The eigenvector topology of the two-resonance nonlinear system can also be revealed by the unique Hamiltonian $H_{3d}$. In the vicinity of an EP, the topological charge 2/*C* in the given complex parameter plane means the geometric phase is 2$\pi$ after *C* cycles around the EP, (*C* is called the eigenvector winding number). In ref [4], the eigenvector winding number *C* is also tied to the splitting of eigenfunctions, thus it can be embedded within the phase rigidity Figure S5(a-d) show the phase rigidities $r=\left( \left\langle\phi_{i}^{R} | \phi_{i}^{R} \right\rangle\right)^{-1}$ for the corresponding cases in Fig. S5(a-d), where we can see $r$ vanishes when approaches the EPs. Fig. S5(e-f) show the corresponding critical behaviors of $r$.

As proved by Bender and Mannheim[10], since all the coefficients of the characteristic polynomial $p\left( \omega\right)$ are real, $H_{3d}$ is *PT* symmetric. Here the general operator *PT* can be written as *PT*=*XK* with *X* obeying *X = X^T^*, *X^2^ = I* and *K* performing complex conjugation. Specifically, for an energy eigenstate that satisfies $H|\left. \phi^{R} \right\rangle=\omega|\left. \phi^{R} \right\rangle$, the antilinearity of the *PT* operator implies that $PTH\left| \left. \phi^{R} \right\rangle=\omega^{*} \right|\left. \phi^{R} \right\rangle=HPT|\phi^{R}\rangle$. If two of the three eigenfrequencies, say, $\omega_{1}$ and $\omega_{2}$ are complex conjugate pair, then the corresponding eigenvectors ($|\left. \psi_{1} \right\rangle$,$|\left. \psi_{2} \right\rangle$) can be related by *PT* operator, i.e., $|\left. \psi_{1}^{R} \right\rangle=PT |\left. \psi_{2}^{R} \right\rangle$. And then the specific form of the *PT* operator is

$$\begin{aligned} PT=(\begin{matrix} \left| \phi_{1}^{R} \right\rangle& \left| \phi_{2}^{R} \right\rangle& \left| \phi_{3}^{R} \right\rangle\end{matrix})\left( \begin{matrix} 0 & 1 & 0 \\ 1 & 0 & 0 \\ 0 & 0 & 1 \end{matrix} \right)K(\begin{matrix} \left| \phi_{1}^{R} \right\rangle& \left| \phi_{2}^{R} \right\rangle& \left| \phi_{3}^{R} \right\rangle\end{matrix})^{-1}\#\left( S21 \right) \end{aligned}$$

Otherwise, all three solutions of Eq. (S$10$) are real, then the 3×3 matrix in Eq. (S$21$) becomes a unity matrix for the *PT* operator. Note here, the *PT* operator we define here is parameter dependent, not the most commonly used PT operator.


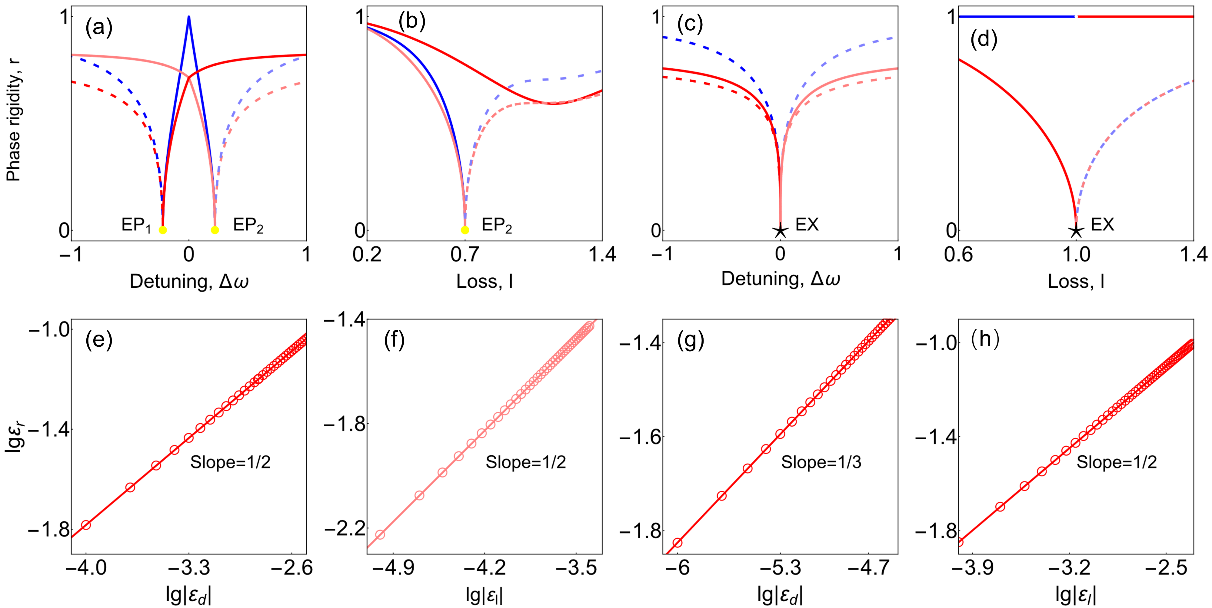


**Figure S5.** (a-d), The phase rigidity $r$ for each steady state as functions of the detuning and loss. $r$ vanishes when approaching the EPs. (e-h), The critical behavior near the corresponding EPs reveals the topological properties of those EPs. The slopes of 1/3 in (G) and of 1/2 in (F). Here the parameters used are the same as the corresponding figures in Fig. S3.

1. **Petermann-factor of stable states and Linear** **PT Hamiltonian with an EX**

In non-Hermitian systems, eigenstates are biorthogonal. The non-orthogonality of eigenstates leads to the increasing of noise when approaching an EP. Thus, the linewidths of modes get broader and therefore reduce the signal-to-noise ratio (SNR) of EP sensors. In the context of optics and photonics, the enhancement of noise due to non-orthogonality is conventionally characterized by the Petermann factor (PF) [11], which is defined as PF$\equiv1/\left| \left\langle\tilde{\psi}^{L} | \tilde{\psi}^{R} \right\rangle\right|^{2}$ with the wavefunctions prenormalized as $\left\langle\tilde{\psi}^{R} | \tilde{\psi}^{R} \right\rangle=\left\langle\tilde{\psi}^{L} | \tilde{\psi}^{L} \right\rangle=1$ At an EP, $\left| \left\langle\tilde{\psi}^{L} | \tilde{\psi}^{R} \right\rangle\right|=0$ and thus PF diverges. If the amplitude of noise is $\sigma$ inside a Hermitian system, then such a noise is enhanced to be PF$\cdot\sigma$ for the non-Hermitian system. To proceed, we calculate the PF for our nonlinear non-Hermitian system. The stable state near the EX is given by

$$\begin{aligned} |\psi\rangle=\left\{ \begin{aligned} \begin{matrix} \frac{1}{\left( \Delta_{\omega}^{2/3}+2 \right)^{1/2}}\left( \begin{matrix} i+\Delta_{\omega}^{1/3} \\ 1 \end{matrix} \right), & \Delta_{\omega}>0, \end{matrix} \\ \begin{matrix} \frac{1}{\left( \left( -\Delta_{\omega} \right)^{2/3}+2 \right)^{1/2}}\left( \begin{matrix} i-\left( -\Delta_{\omega} \right)^{1/3} \\ 1 \end{matrix} \right), & \Delta_{\omega}<0. \end{matrix} \end{aligned} \right.\#\left( S22 \right) \end{aligned}$$

Thus, the PF is

$$\begin{aligned} PF=1-\left( 4+\left| \Delta_{\omega} \right|^{\frac{2}{3}} \right)^{-1}+\left| \Delta_{\omega} \right|^{-\frac{2}{3}}\approx\left| \Delta_{\omega} \right|^{-\frac{2}{3}}.\#\left( S23 \right) \end{aligned}$$

At the EX point, the splitting of eigenfrequency (signal) is also enhanced. This signal-enhancement-factor (SEF) is given by $\left| \partial{\omega/\partial\Delta}_{\omega} \right|^{2}\propto\left| \Delta_{\omega} \right|^{-4/3}$. With the PF and SEF at hand, we can calculate the minimum detectable signal, which is proportional to (PF/SEF)^1/2^ $\propto\left| \Delta_{\omega} \right|^{1/3}$. Thus, SNR indeed is dramatically improved when approaching the nonlinear “EX” in our system.

In our system, the noise is suppressed by the feedback mechanism of the saturable gain. This can be seen from the envelope of Re[$\psi_{A}$] in Fig. 3(d and g) where the fluctuation of the envelope is much less than the noise (the blue lines). The fact is that the derivation from the stable states introduced by the noise is unstable as can be seen from the Lyapunov exponents as plotted in Fig. S2. At $\Delta_{\omega}=0$, the PF diverges at exactly the EX point according to Eq. (S$23$), and then the enhanced noise is too strong to be compensated by the feedback mechanism. However, as the gain coefficient shifts away from the EX condition due to the presence of noise, the PF decrease rapidly into a finite value as the eigenstates no longer coalesce. As a result, the amplitude of noise at $\Delta_{\omega}=0$ remains at a finite value.

To obtain the SNR of the system from dynamics, we set D = ζ= 0.3 and record $\psi_{A}$ within a finite period $0\leq t\leq$ *T* for 100 independent simulations. After that, we perform the fast Fourier transform (FFT) and plot the average center frequency and line width versus $\Delta_{\omega}$ in Fig. S6. As shown in Fig. S6, the center frequency agrees perfectly with the frequencies of steady states solved from Eq. (2) in the main text. The noise introduces a minor frequency shift which is less than $2‰$ over the parameter of interest. Due to the finite simulation time, there is an uncertainty ($\propto1/T$) when we fit the line width and this uncertainly emerges as background noise as shown with the cyan background in Fig. S6(b). Near $\Delta_{\omega}=0$, the line width remains at a finite value. Away from EX, the line width decreases quickly into the cyan background as can be seen from the enlarged plot in Fig. S6(c). The decreasing of line width is caused by the fact that the white noise we add can be compensated by the feedback mechanism of the saturable gain. With the center frequency and corresponding line width obtained, we can then plot the SNR^-1^ versus $\Delta_{\omega}$ in Fig. 1(d) using Eq. (6).


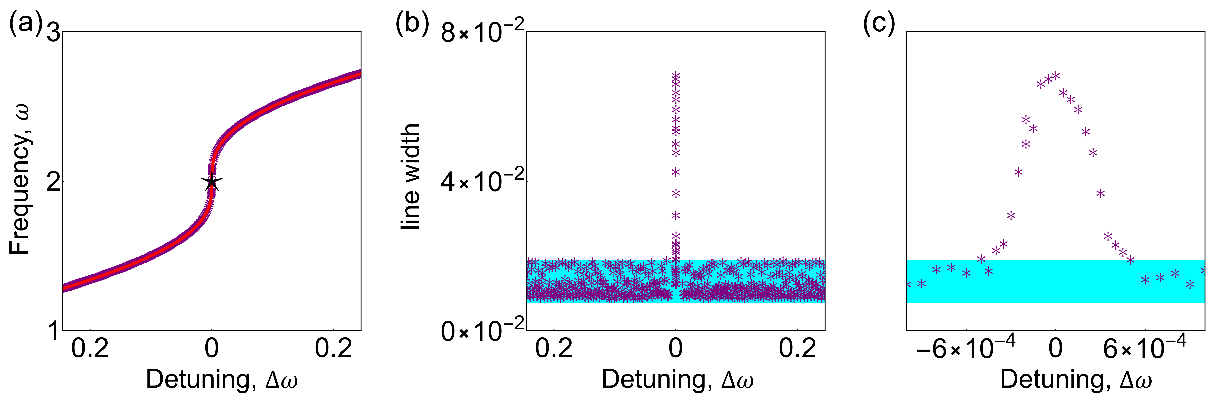


**Figure S6.** (a), The retrieved center frequency (purple asterisks) together with the steady state eigenfrequencies from Eq. (2) (red solid lines). (b), The average line width versus $\Delta_{\omega}$, here the cyan background (from $2\pi$*/T* to $5\pi$*/T*) represents the line width uncertainty introduced by the finite simulation time *T*. (c) zooms in around $\Delta_{\omega}=0$. In these simulations, *T* = 2^23^$\times$10^-4^, D = ζ= 0.3 and the average is performed over 100 independent simulations. All the other parameters are the same as Fig. 3.

For comparison, we also consider the dynamics around the EX of a linear PT symmetric three-resonator Hamiltonian $H_{ex}$ adapted from Ref. [4].

$$\begin{aligned} i\frac{d}{dt}\left( \begin{matrix} \psi_{A} \\ \psi_{N} \\ \psi_{B} \end{matrix} \right)=\kappa\left( \begin{matrix} \omega_{0}+i\sqrt{2}\left( 1+\delta g \right)+i \zeta\xi_{g} & 1 & 0 \\ 1 & \omega_{0}-\Delta_{\omega} & 1 \\ 0 & 1 & \omega_{0}-i\sqrt{2}\left( 1+\delta g \right) \end{matrix} \right)\left( \begin{matrix} \psi_{A} \\ \psi_{N} \\ \psi_{B} \end{matrix} \right)+D\left( \begin{matrix} \xi_{e} \\ 0 \\ 0 \end{matrix} \right) ,\#\left( S24 \right) \end{aligned}$$

where $\omega_{0}$ is the onsite eigenfrequency, $\Delta_{\omega}$represents the detuning, $\kappa$ denotes the strength of coupling (set as 1 for simplicity for the discussion below), and $\delta g$is the loss or gain magnitude. Similar as the main text, $\xi_{g,e}(t)$ is the Gaussian white noise with mean 0, and $\zeta$ and D represent the corresponding standard deviations. The Hamiltonian $H_{ex}$ exhibits an EX at $\Delta_{\omega}=\delta g=0$ where three eigenstates coalesce into one. Figure S7 shows the real part of eigenfrequencies $Re[\omega]$, the imaginary part of eigenfrequencies $\mathrm{Im}\left[ \omega\right]$, phase rigidity *r*, and the inverse of PF, (PF)^-1^, along both the $\delta g$ direction ($\Delta_{\omega}=0$) and the $\Delta_{\omega}$ direction ($\delta g=0$). The critical exponent of$Re[\omega]$, $Im[\omega]$, and *r* have been demonstrated in Ref. [[4]]. Here we focus on the $\Delta_{\omega}$ direction along which $\omega\approx\sqrt[3]{-2\Delta_{\omega}}$ near the EX, and thus $\mathrm{SEF}$ is proportional to $\left| \Delta_{\omega} \right|^{-4/3}$. Meanwhile, the eigenstates near the EX is given by $\left( -1+i\sqrt{2}\sqrt[3]{-2\Delta_{\omega}},i\sqrt{2}+\sqrt[3]{-2\Delta_{\omega}},1 \right)^{\text{T}}$ and then PF is also proportional to $\left| \Delta_{\omega} \right|^{-4/3}$. It is clear that the improved SEF (responsivity) is precisely compensated by the enhanced noise as the PF exhibits the same critical component. As a result, the SNR remains at a finite value (not diverges) when approaching the EX in a linear system as can be seen from the green dashed line in Fig. 1(c). Hence, we can conclude that the SNR near the EX is not expected to improve based on this specific measurement scheme.


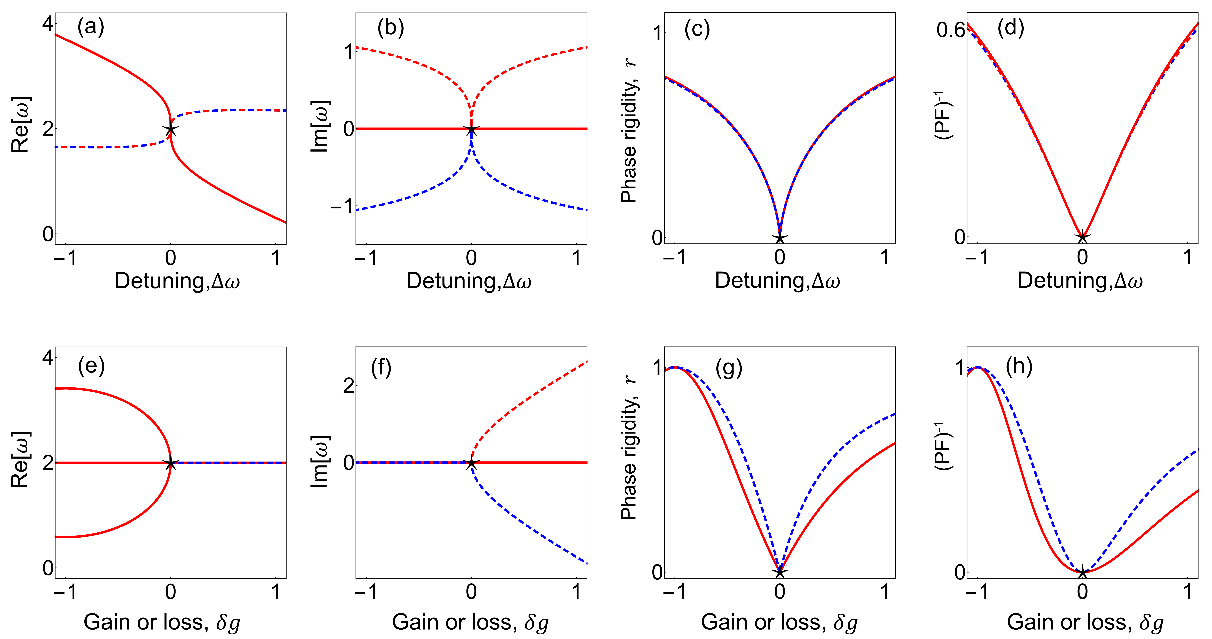


**Figure S7.** Real part (a, e) and imaginary part (b, f) of the eigenfrequencies of $H_{ex}$ versus the detuning $\Delta\omega$ and gain or loss magnitude $\delta g$. (c, g) The phase rigidity $r$ vanishes when approaching the EX. (d, h) The Petermann-factor (PF), a measure of nonorthogonality, gives the noise enhancement near the EX. $\omega_{0}=2$ and $\kappa=1$ in (a-h), $\delta g=0$ in (a-d), and $\Delta_{\omega}=0$ in (e-h).

1. **Negative resistance.**

A negative resistor is opposite to an ordinary resistor which means that when a positive voltage is applied to the terminal, a proportional negative current comes out of the terminal. A negative resistor can feed power to the circuit. In our system, it is realized with an operational amplifier (op-amp). Figure S8(a) illustrates how a simple linear op-amp can be configured to achieve negative resistance. In the linear unsaturation region, the op-amp can be regarded as an ideal one, and the voltage $V_{1}$ on node ① is equal to the voltage on node $②$. Meanwhile, the current passing through the resistor $R_{g}$, $I_{Rg}=V_{1}/R_{g}$, must also pass through the diodes to node $③$, i.e., no current passes through node $②$. Thus, the voltage on node $③$, $V_{③}$ is given by

$$\begin{aligned} V_{③}=V_{1}+\frac{V_{1}}{R_{g}}R_{D},\#\left( S25 \right) \end{aligned}$$

where$R_{D}$ is the resistance of the two diodes composite (current can pass through in both directions). As will discuss later on, $R_{D}$ is a function of $V_{1}$ and here for simplicity, we do not write down the $V_{1}$ dependence explicitly. Then the current passing through the resistor $R_{1}$ is given by

$$\begin{aligned} I_{R_{1}}=\frac{{V_{1}-V}_{③}}{R_{1}}=-\frac{V_{1}}{R_{g}R_{1}}R_{D}, \#\left( S26 \right) \end{aligned}$$

Here the “$-$” sign means the current $I_{R_{1}}$ has a direction opposite to the voltage at node ①. Hence from the perspective of the red node in Fig. S8(a), the effective negative resistance ($R_{n}$) of the whole system inside the dashed box is

$$\begin{aligned} R_{n}=\frac{V_{1}}{I_{R_{1}}}=-\frac{R_{g}R_{1}}{R_{D}}. \#\left( S27 \right) \end{aligned}$$

Figure S8(b) shows a typical volt-ampere characteristic curve of the two diodes composite. It implies that the resistance of the two-diode composite $R_{D}$ actually depends on the current passing through it as shown by the green solid line in Fig. S8(b). Seeing that the current passing through the diode $I_{Rg}$ is a function of $V_{1}$, the resistance $R_{D}$ is a function of $V_{1}$ and so is $R_{n}$ according to Eq. (S$27$). Over the range of interest, $R_{D}$ should also be monotonic a decreasing function of ${|V}_{1}|$ and hence $R_{n}$ should be an increasing function of ${|V}_{1}|$. To verify the rationality of the above approximation, we perform simulations with Ltspice [12] based on the circuit model in Fig. S8(a). We start the simulation with a small initial voltage (1 μV) at node ① to kick start oscillations and Fig. S8(c) shows the evolution of $V_{1}$ till a steady state is reached. At the steady state, $V_{1}$ oscillates at the resonance frequency of the LC resonator. The envelope of $V_{1}$ reaching its maximum after some time indicates the presence of a saturated gain. Noting that the normal resistor $R$ and a negative resistor connected in parallel in Fig. S8(a), and hence the total resistance of such a LC circuit is $RR_{n}/(R+R_{n})$. When $V_{1}$ is small, $R_{n}$ is a small negative number, then the total resistance is negative and thus $V_{1}$ increase with time. Eventually, $R+R_{n}$ approaches zero and $V_{1}$ reaches a saturated steady value. In other words, we can build a one to one correspondence between the steady value of $V_{1}$ and $R_{n}=-R$ from the simulations considering realistic op-amp and diodes. The solid line and circles show $|R_{n}|$ versus $V_{1}$ according to Eq. (S$27$) and simulations, respectively. The deviations between Eq. (S$27$) and simulation mainly come from the influence of the unideal op-amp. However, the monotonic increasing of $|R_{n}|$ as a function of $V_{1}$ still preserves which will introduce nonlinear saturable gain in our two-resonator circuit.


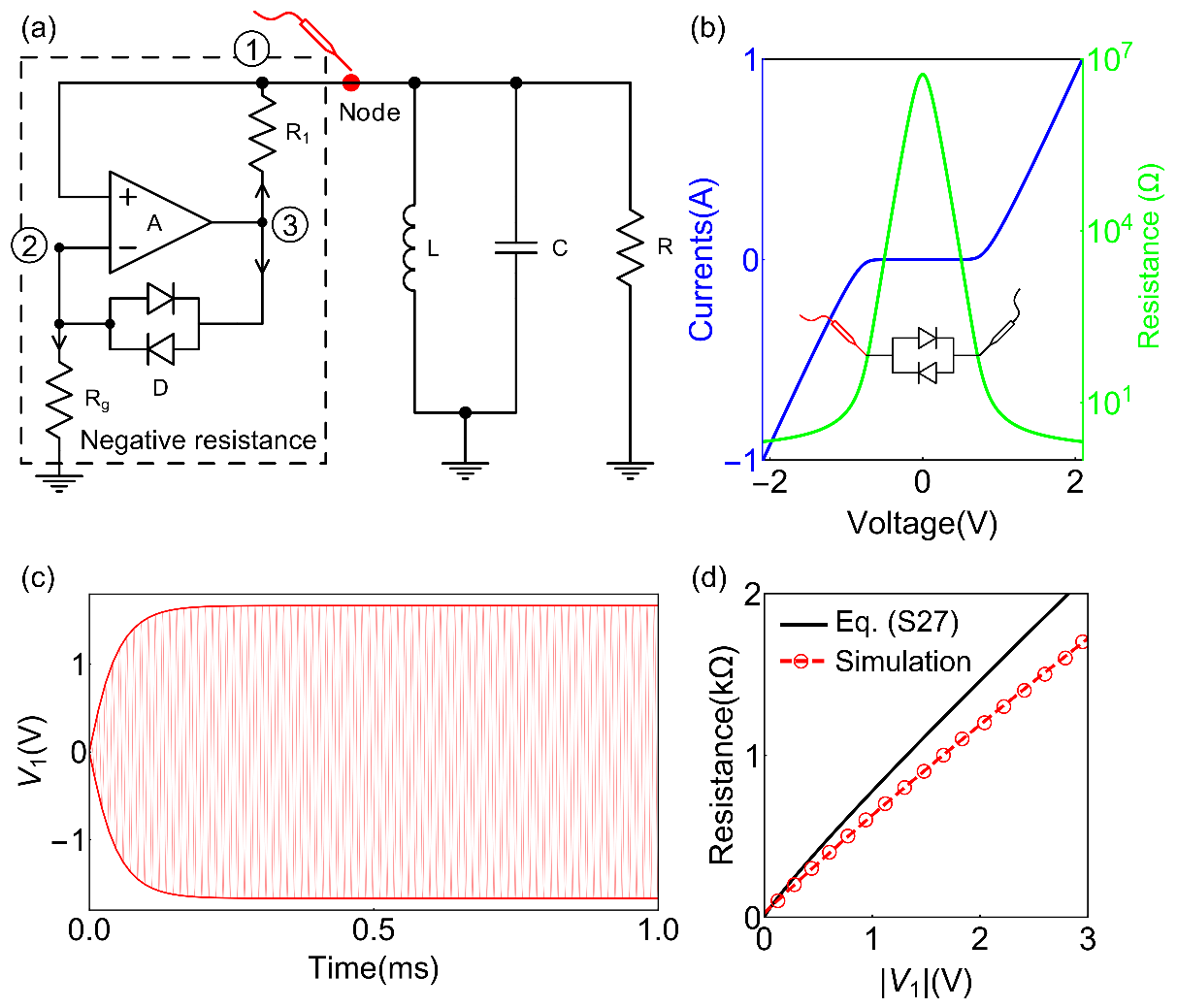


**Figure S8.** (a) A LC circuit with a normal resistor R and an effective negative resistor (marked by the black dashed box) connected in parallel. The negative resistor consists of two normal resistors $R_{1}$ and $R_{g}$, an op-amp and two diodes. (b) The *I-V* characteristic curve (blue) and the resistance (green) of the two diodes composite used (Onsemi BAV99L). The inset shows the measurement setup. (c) Evolution of $V_{1}$ on the red node with a small kick start voltage $V_{1}=$1 μV, where the solid red lines highlight the envelope. (d) $|R_{n}|$ as a function of $V_{1}$, where the black line and the red circles are obtained with Eq. (S$27$) and from the simulations, respectively. In both (c and d), $R_{g}=5.1 k\Omega$, $R_{1}=400 \Omega$,$L=220uH$ and $C=18 nF$ are used, the serious number of the op-amp is (TI LM7171). We set $R=1k \Omega$ in (c) and change the value of *R* in simulations to find the relation between $V_{1}$ and $|R_{n}|$ in (d).

1. **Derivation of the coupled-mode equations.**

In this section, we derive Eq. (1) in the main text from Kirchoff’s equations for the circuit in Fig. 3(a). From the analysis in Sec. 3, we can see that the effects of the circuit elements inside the dashed box in Fig. 3(a) can be effectively replaced by an effective negative resistance $R_{A}\left[ V_{A} \right]=-R_{g}R_{1}/R_{D}\left[ V_{A} \right]$. Assume that the circuit is working with a time dependency of $e^{-i\omega t}$, Kirchoff’s equations are

$$\begin{aligned} I_{L,A}+\frac{V_{A}}{R_{A}}-i\omega CV_{A}-i\omega C_{c}\left( V_{A}-V_{B} \right)=0, \\ I_{L,B}+\frac{V_{B}}{R_{B}}-i\omega CV_{B}-i\omega C_{c}\left( V_{B}-V_{A} \right)=0,\#\left( S28 \right) \end{aligned}$$

where $I_{L,X}$, $R_{X}$ and $V_{n}=-i\omega L_{X}I_{L,X}$ with $X=A, B$ are the currents flowing through the inductors, the resistances and voltages, respectively. Eliminate $I_{L,X}$and set $\omega_{A,B}=1/\sqrt{L_{A,B}C}$ to represent the resonant frequency of the uncoupled resonator, Eq. (S$28$) can be reformulated in a matrix form as

$$\begin{aligned} \left( \begin{matrix} \frac{-i}{R_{A}C}+\frac{\left( \omega_{A}^{2}-\omega^{2} \right)}{\omega}-\frac{C_{c}}{C}\omega& \frac{C_{c}}{C}\omega\\ \frac{C_{c}}{C}\omega& \frac{-i}{R_{B}C}+\frac{\left( \omega_{B}^{2}-\omega^{2} \right)}{\omega}-\frac{C_{c}}{C}\omega\end{matrix} \right)\left( \begin{matrix} V_{A} \\ V_{B} \end{matrix} \right)=0. \#\left( S29 \right) \end{aligned}$$

If we further assume $C_{c}/C\ll1$ and $\left| \omega_{A,B}-\omega\right|\ll\omega$ (working frequency near the resonance frequency $\omega_{A,B}$, Eq. (S$29$) becomes

$$\begin{aligned} \left( \begin{matrix} \omega_{A}-\omega-\frac{i}{2R_{A}C} & \frac{C_{c}}{2C}\omega_{B} \\ \frac{C_{c}}{2C}\omega_{B} & \omega_{B}-\omega-\frac{i}{2 R_{B}C} \end{matrix} \right)\left( \begin{matrix} V_{A} \\ V_{B} \end{matrix} \right)=0. \#\left( S30 \right) \end{aligned}$$

Compare with Eq. (1), we can see that the resonance frequency, the coupling, the loss rate and the gain are given by $\omega_{A,B}=1/\sqrt{L_{A,B}C}$, $\kappa=\omega_{B}C_{c}/2C$, $l=1/2R_{B}C$and $g\left[ V_{A} \right]=R_{D}\left[ V_{A} \right]/2 R_{g}R_{1}C$, respectively. As can be seen in Fig. S8(b), $R_{D}\left[ V_{A} \right]$ is a monotonic decreasing function of $V_{A}$, thus such a system should also exhibit saturable gain with the increasing of $V_{A}$.

1. **Circuit elements on the PCB.**

In this section, we provide the details of circuit elements on the PCB. The upper panel of Fig. S9 shows a photo of the PCB used in our experiments, wherein different functional regions are basically located according to the circuit in Fig. 3(a) as outlined by the dashed box. The lower panels of Fig. S9 show the zoom in the photos of a few representative elements. From ① to ⑦ are inductor (Murata MDH12577C), resistor $R_{g}$ = 5.1 kΩ (PTFR0603B5K10N9), diode (Onsemi BAV99L), op-amp (TI LM7171), capacitor (Murata GRM21B5C1H183JA01L), variable resistor (3296W-1-502LF) and barrier terminal block, respectively. Here the barrier terminal blocks have two nodes and each of which can be used for measuring the voltage and connecting the circuit element as designed.


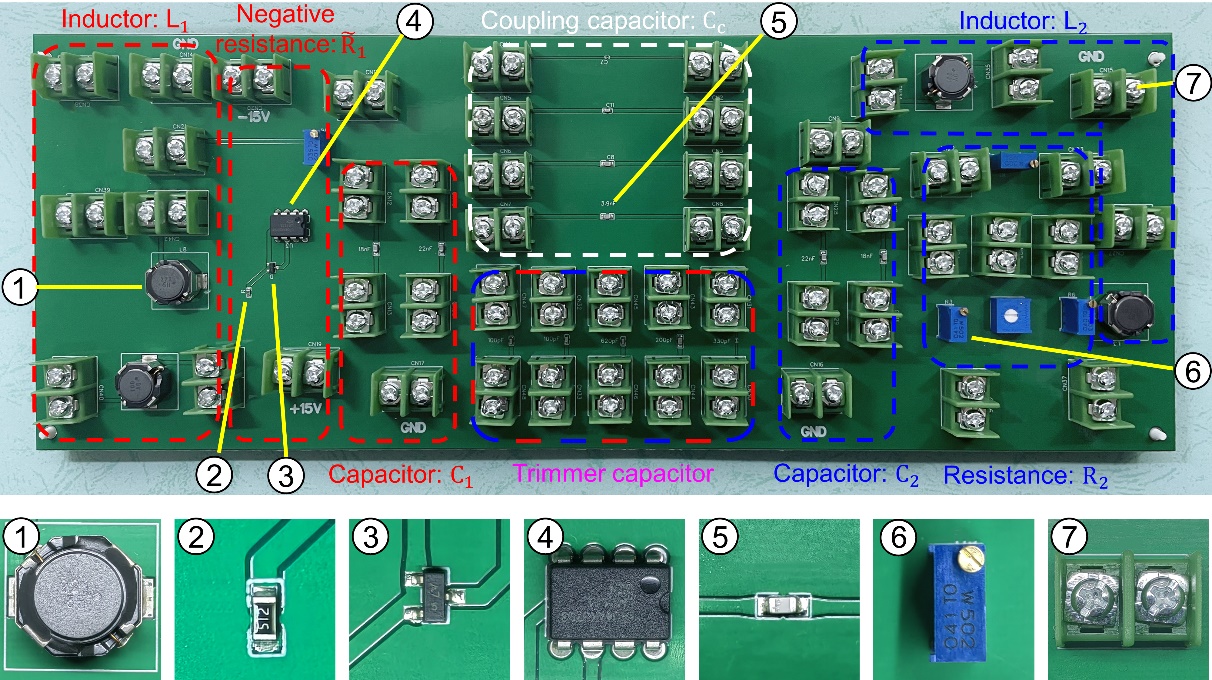


**Figure S9.** Details of circuit elements on the PCB. ① inductor, ② resistor, ③ diode, ④ operational amplifier, ⑤ capacitor, ⑥ variable resistor, ⑦ barrier terminal block.

To continuously tune the loss and frequency detuning, we also introduce variable resistors and a homemade continuously tunable inductor as shown in Fig. S9. Figure S10(a) shows an enlarged photo of one variable resistor and Fig. S10(b) shows the details of the tuning mechanism. There is an adjustment screw on top of the variable resistors and by screwing the screw, the corresponding resistance changes. The inductor element $L_{B}$ consists of two inductors connected in series, one with inductance around 220uH (Murata MDH12577C-221MA) and another one about 10uH (Murata MDH12577C-100MA). The inductor $L_{A}$ also contains another homemade continuously adjustable inductor such that the inductance can cover the range of interest. Fig. S10(c) and (d) show a photo and the sketch of the homemade tunable inductor used, respectively. The inductance depends on the distance between the two blue hexagon nuts. When the distance between the hexagon nuts increases, the copper wires (brown) turns spread, and hence the inductance decreases as shown in Fig. S10(e).


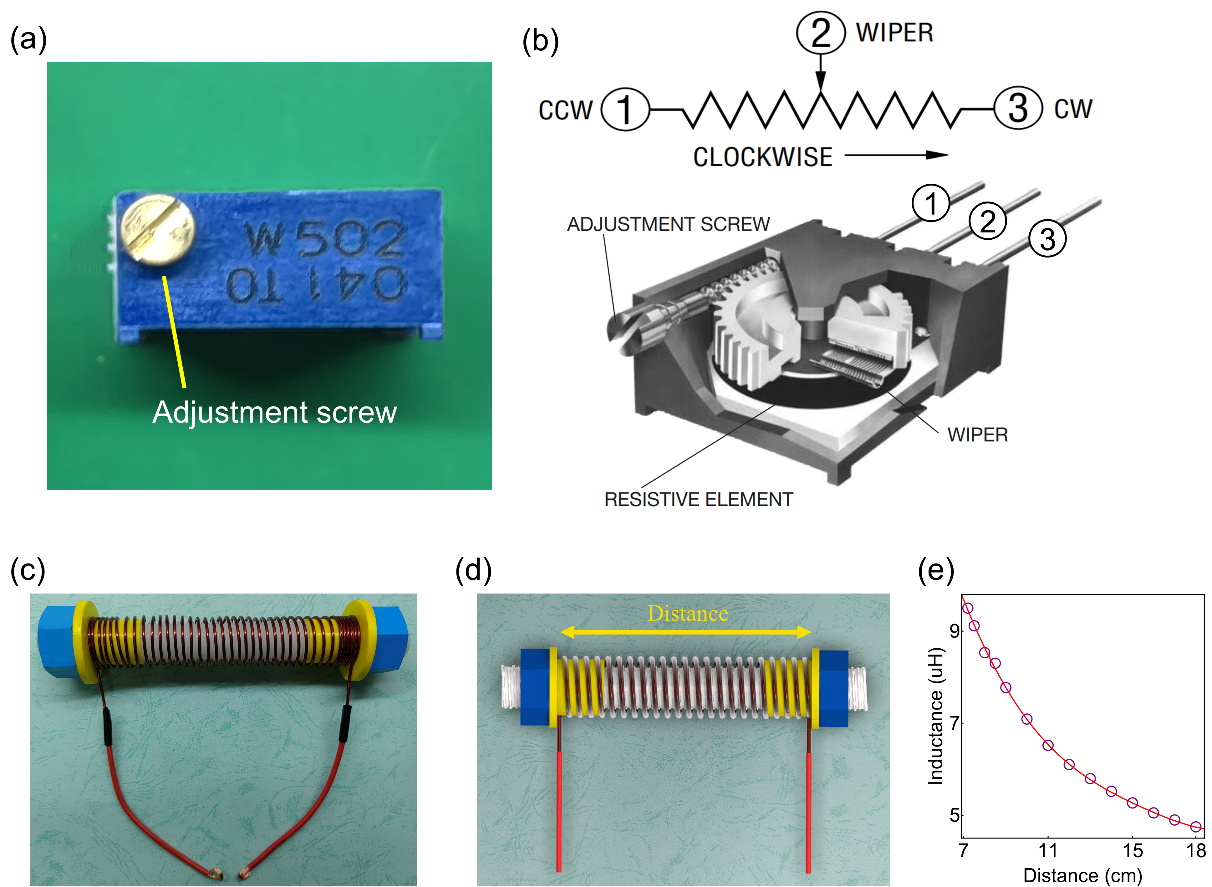


**Figure S10.** (a) Photo of a variable resistor. (b) The internal structure of the variable resistor [13]. The resistance can be controlled by the adjustment screw. (c) Photo of the homemade tunable inductors. It is wound with 39 turns of #15 copper wire on a PVC cylinder with diameter 2 cm and length 19 cm. (d) Sketch shows the structure of the tunable inductor, where the distance between two blue hexagon nuts determines the inductance. (e) Measured inductance as a function of the distance defined in (D), where magenta circles show the measured data and the red line is just for guidance.

1. **Switching between stable states.**

In this section, we simulate the switching between bistable states using external driving. The simulation is performed with the circuit in Fig. 3(a) using LTspice. $V_{A}$ is initialized with a small voltage (1 μV) to kick-start oscillations. Due to the presence of a saturable gain, the magnitudes of $V_{A}$ and $V_{B}$ oscillate and increase until one of the stable states is reached at around $0.7 ms$. This stable state has a resonant frequency of 78.6 $\mathrm{kHz}$ (as can be seen from Fourier spectra in the inset), $\left| V_{A} \right|$=2.273$V$ and $\left| V_{B} \right|$=2.155$V$ with $\left| V_{A}/V_{B} \right|^{2}>1$. At $1.5 ms$, we enforce an external driving signal of 4V and 63$\mathrm{kHz}$ on $V_{A}$ for 70 cycles. (In experiments, this step is realized with an arbitrary waveform generator.) Under the driving signal, $V_{A}$ and $V_{B}$ start oscillation and get steady at around $2.3 ms$. At $2.6 ms$, we remove the driving signal. Once again, $V_{A}$ and $V_{B}$ start oscillation and reach another stable state at around $4.8 ms$. This stable state is different from the previous one. Such a state has a resonant frequency of 67.6 $\mathrm{kHz}$, $\left| V_{A} \right|$=1.445$V$ and $\left| V_{B} \right|$=1.584$V$ with $\left| V_{A}/V_{B} \right|^{2}<1$. Thus, we realize switching between bistable states in our system by enforcing an external driving signal. Note here, we purposely choose the driving frequency and strength such that the final state falls into the other targeting stable state. The above scheme is implemented in experiments to enforce switching between two states such that both states can be measured.


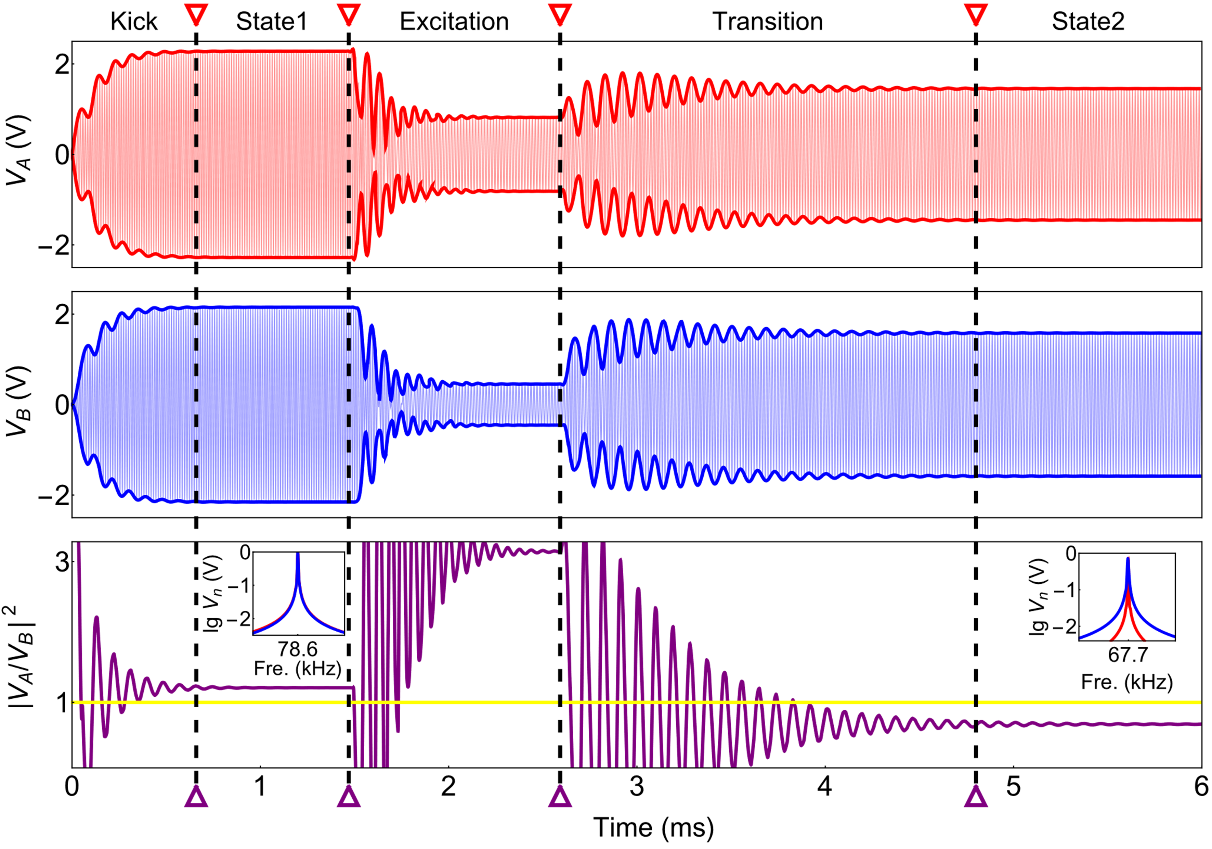


**Figure S11.** The red, blue and purple lines show the dynamics of $V_{A}$, $V_{B}$ and $\left| V_{A}/V_{B} \right|^{2}$, respectively. The simulation is started with an initial small potential (1 μV) for $V_{A}$. At $1.5 ms$, an external driving signal of 4V and 63$\mathrm{kHz}$ is added on $V_{A}$ for 70 cycles and then removed at $2.6 ms$. The insets in the lowest panel correspond to the Fourier transform of $V_{A}$ (red) and $V_{B}$ (blue) between [0.7 ms, $1.5 ms$] (left) and [4.8 ms, $6.0ms$] (right). In the simulation, $R_{g}=5.1 k\Omega$, $R_{1}=400 \Omega$, $L_{A}=215uH$ and $L_{B}=220uH$ (Murata MDH12577C and the series resistance *R_LB_ =*4$\Omega$), $C=18.5 \mathrm{nF}$, $C_{c}$=3.85 nF, $R_{2}$=2k$\Omega$, and the parameters of the op-amp (TI LM7171) and diodes (Onsemi BAV99L) are adapted from the database. The parameters used here are consistent with those extracted from the experiments.

1. **Measured eigenfrequencies versus circuit parameters.**

In this section, we provide measured resonance frequencies and $\left| V_{A}/V_{B} \right|^{2}$ values for the stable steady states as functions of $R_{B}$ and inductor $L_{A}$, which are the tuning knobs in the experiments. $R_{B}$ is a variable resistor with the detailed structure shown in Fig. S10. $L_{A}$ consists of a homemade variable resistor connected in series with another standard inductor (Murata MDH12577C-221MA) with inductance measured to be 204uH (at 72kHz and 1V). The markers in Fig. S12 (a-d) and (e-h) show the measured resonance frequencies and the corresponding $\left| V_{A}/V_{B} \right|^{2}$ as we change $R_{B}$ and $L_{A}$, respectively, where the values of $R_{B}$ and $L_{A}$ are also measured experimentally with a precision LCR meter (TH2829C). Figure S12 (a-b) and (e-f) correspond to the case which exhibits two EPs, and Fig. S12 (c-d) and (g-h) focus on the EX. The solid lines in Fig. S12 are obtained from numerical simulations with the red and blue lines representing the stable and unstable steady states, respectively. Meanwhile, the circuit has unavoidable parasitic capacitance. To cancel the effect of this parasitic capacitance, the values of $L_{A}$ used in simulations have been shifted upward relative the to measured values by 2.2$\mu H$ in Fig. S12(a, e) and 1.4 $\mu H$ in Fig. S12 (c, g). The detailed reasoning will be discussed in the error analysis section later.


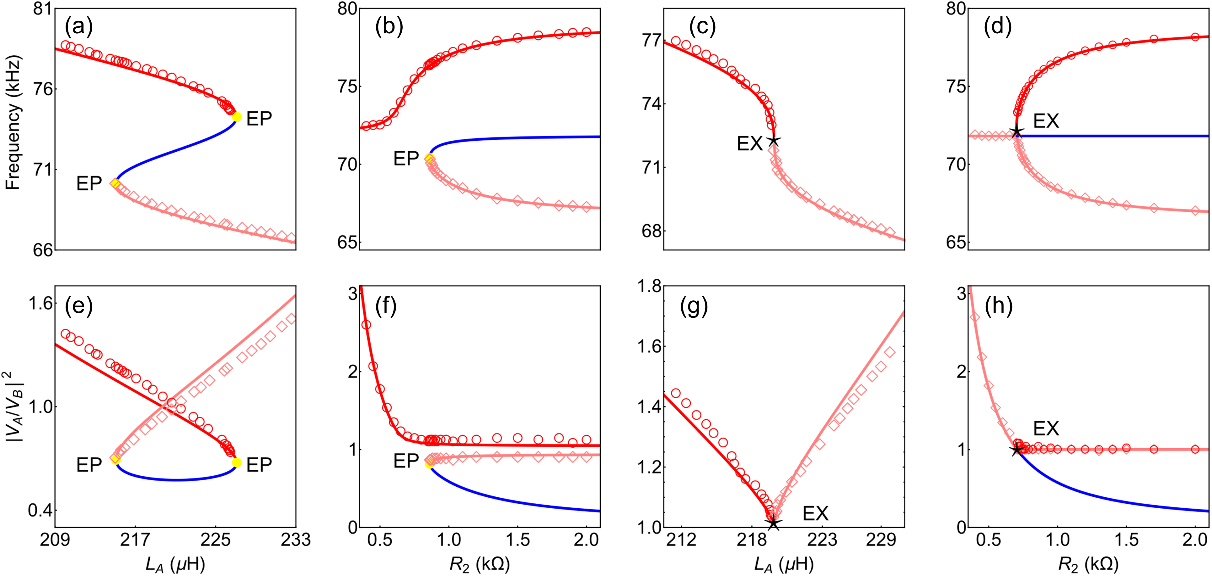


**Figure S12.** At detuned system, the steady-state frequency as a function of the inductance $L_{A}$ (a) and resistance $R_{2}$ (b). $\left| V_{A}/V_{B} \right|^{2}$ as a function of the inductance $L_{A}$ (e) and resistance $R_{2}$ (f). At zero detuning, the steady-state frequency as a function of the inductance $L_{A}$ (c) and resistance $R_{2}$ (d). $\left| V_{A}/V_{B} \right|^{2}$ as a function of the inductance $L_{A}$ (g) and resistance $R_{2}$ (h). The estimated experimental errors are smaller than the marker sizes and are hence not explicitly shown. The influence of the parasitic capacitance in the circuit has been taken into consideration here (Details provided in the error analysis section). $R_{2}$*=*1k$\Omega$ in (a and e),$L_{A}=218uH$ in (b and f), $R_{2}$*=*708$\Omega$ in (c and g), $L_{A}=220uH$in (d and h). Except for the above parameters, all other parameters: $L_{B}=220uH$,$C=18.5 \mathrm{nF}$, $C_{c}$=3.85 nF, and the inherent series resistance *R_inh_* =4$\Omega$. (defined later in the error analysis section) are the same for all those simulations above.

1. **Experimental error analysis.**

Realistic circuit elements used in the experiments such as the capacitors and inductors are not ideal. For example, an inductor can be accompanied by a parasitic parallel resistance (*R_par_*), a series resistance (*R_ser_*), and a parallel shut capacitance (*C_par_*) as shown in Fig. S13(a). The situation is similar for a capacitor with an equivalent circuit shown in Fig. S13(b), where we also include equivalent series inductance (*L_ser_*) and shunt resistance across it (*R_Lsh_*). In our experiments, we carefully choose the capacitors (Murata GRM21B5C1H183JA01L) and inductors (Murata MDH12577C) such that those elements can be regarded as capacitors or inductors in series with inherent resistors (labeled as $R_{C}$ or $R_{L}$) as shown in Fig. S13(c). In other words, the unwanted *R_par_*, *C_par_*, *L_ser_* and *R_Lsh_* are negligible small in the experiments.

Consider a resonance circuit as shown in Fig. S13(c), assume the circuit is working with a time dependency of $e^{-i\omega t}$, the Kirchoff’s equation is given by

$$\begin{aligned} \frac{1}{-i\omega L+R_{L}}+\frac{1}{R}+\frac{1}{\frac{-1}{i\omega C}+R_{C}}=0. \#\left( S31 \right) \end{aligned}$$

In the experiments, $R_{C}$ and $R_{L}$ are much smaller than $R$ with typical values of $R_{C}/R$and $R_{L}/R$ less than 0.5%. Thus we can take the approximation $R_{C}/R\ll1$ and $R_{L}/R\ll1$, and Eq. (S$31$) reduces to

$$\begin{aligned} \omega^{2}+i\left( \frac{1}{CR}+\frac{R_{C}+R_{L}}{L} \right)\omega-\frac{1}{CL}\approx0. \#\left( S32 \right) \end{aligned}$$

Hence the circuit in Figure S13(c) can be described by an equivalent circuit shown in Fig. S13(d) with an equivalent resistance $R_{\mathrm{eff}}$ satisfying

$$\begin{aligned} \frac{1}{R_{\mathrm{eff}}}=\frac{1}{R}+\frac{C\left( R_{C}+R_{L} \right)}{L}. \#\left( S33 \right) \end{aligned}$$

Following the derivation in Sec. 5, we can see that the loss rate *l* of a cavity is shifted by $\left( R_{C}+R_{L} \right)/2L$ due to the presence of $R_{C}$ and $R_{L}$. In the experiments, the barrier terminal blocks and the connection wires also introduce small resistances, and such the resistances can be treated similarly as $R_{C}$ and $R_{L}$. All these resistances discussed above can be taken into consideration by an effective $R_{\mathrm{eff}}$ which shifts by *R_inh_* relative to the measured resistance of the resistor. This additional *R_inh_* shifts the positions of EAs and EX in the parameter space along the loss direction. In the simulations, the effect of *R_inh_* is taken into consideration by changing the value of *R_L_* accordingly for simplicity.


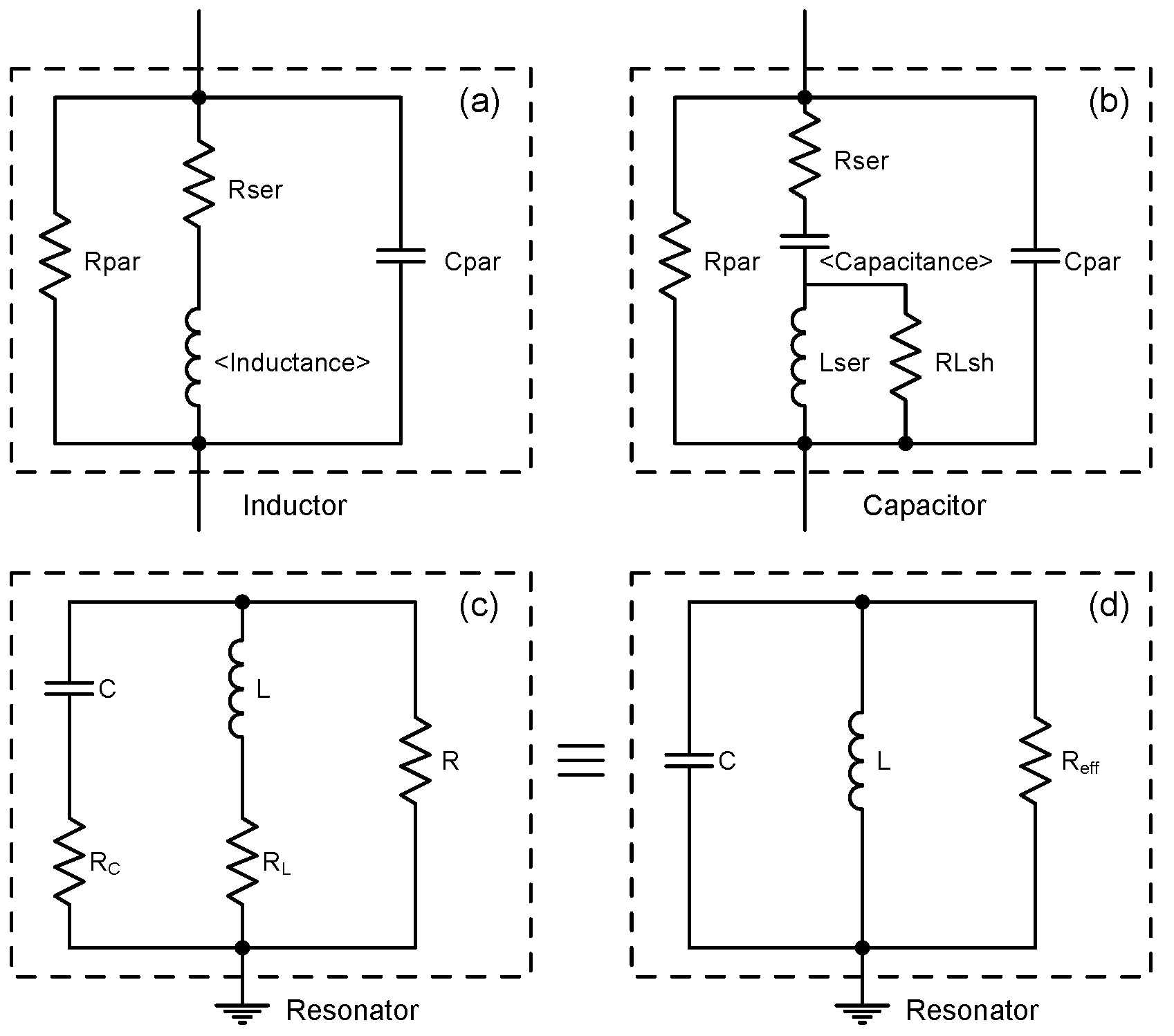


**Figure S13.** The circuit diagrams of a realistic inductor (a) and a realistic capacitor (b). We carefully choose the capacitors and inductors such that those circuit elements in the experiments can be regarded as capacitors or inductors only in series with resistors (labeled as $R_{C}$ and $R_{L}$) as shown in (c), where a typical LRC circuit is considered. $R_{C}$ and $R_{L}$ are much smaller than *R* in the experiments, and hence the LRC circuit in (c) is equivalent to the circuit in (d), where the resistor *R* is replaced by an effective resistance $R_{\mathrm{eff}}$.

In principle, the inductance and capacitance of circuit elements depend on the working frequency and voltage. Figure S14 shows the measured inductance of $L_{B}$ and the measured capacitance C versus the applied voltage and working frequency. Among the frequency of interest, the change of inductance is less than 0.4%, which is pretty small. The inductance change is relatively faster as a function of voltage. On the other hand, the variations of capacitance are negligible small in the experiments as shown in Fig. S14 (c and d). To minimize the effect of voltage dependence, we slightly vary the resistance of $R_{1}$ (450 ±$50 \Omega$) in Fig. 3(a) to maintain $V_{B}\approx1V$ in all the measurements, such that the change of $L_{B}$ can be ignored in the experiments. On the other hand, different steady states have different ratios of $\left| V_{A}/V_{B} \right|^{2}$ according to Fig. S12. Since we have fixed $V_{B}$, the change of $L_{A}$ ($\sim1\mu H$) due to the change of $V_{A}$ is unavoidable. The correction of $L_{A}$ will be discussed later below.


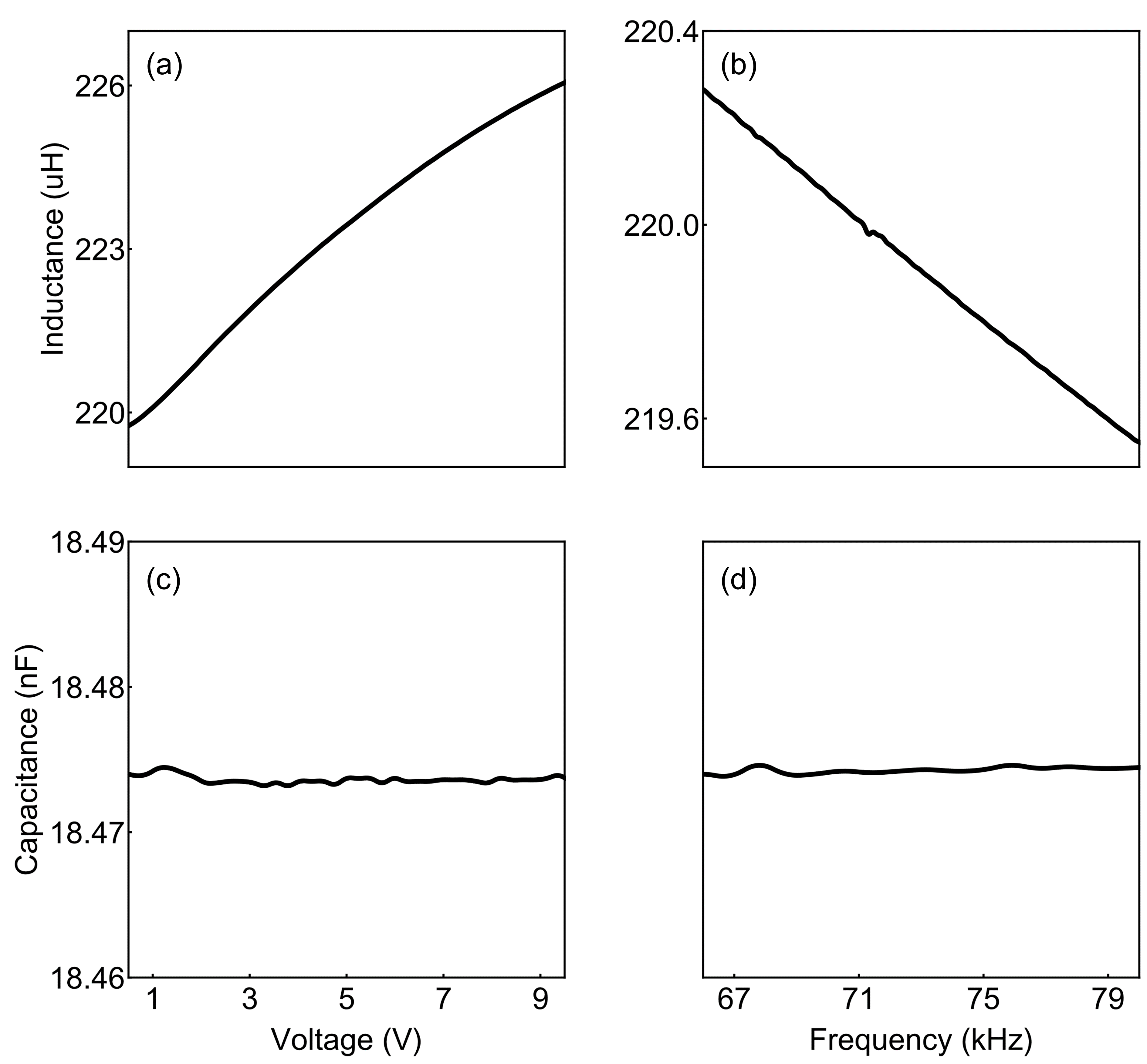


**Figure S14.** Measured inductance $L_{B}$ versus voltage (a) and frequency (b), and measured capacitance *C* (for the lossy resonator) versus voltage (c) and frequency (d).

The negative resistance component as shown in Fig. S15(a) unavoidably introduces unwanted parasitic capacitance *C_pn_*. *C_pn_* mainly comes from the junction capacitance of the diodes and the parasitic capacitance of the op-amp. *C_pn_* can be revealed by measuring the voltage and current simulatively on node_1_ (the red dot in Fig. S15(a)). The solid red and green lines in Fig. S15(c) sketch the typical voltage and current curves, respectively. Ideally, there should be a $\pi$ phase difference between the voltage and current since the resistance is negative. However, due to the unwanted parasitic capacitance *C_pn_*, the phase shift is not exact $\pi$, i.e., the voltage and current curves do not cross at zero. Here, for demonstration purposes, we exaggerate the error of phase shift (we use 1N5817 instead of BAV99L for diodes) and the typical relative error in the experiment is $<1\%$. Such an effect of the parasitic capacitance can be canceled by an offset inductor $L_{o}$ as shown in Fig. S15(b). With $L_{o}$, the phase difference between the current (green dashed line in Fig. S15(c)) and the voltage can return $\pi$. In the experiment, $L_{o}$ is much larger than $L_{A}$ with typical values estimated to be $L_{A}/L_{o}\sim1\%$. Since $L_{o}$ is in parallel with $L_{A}$, the presence of $L_{o}$ slightly decrease the effective inductance in the experiments.


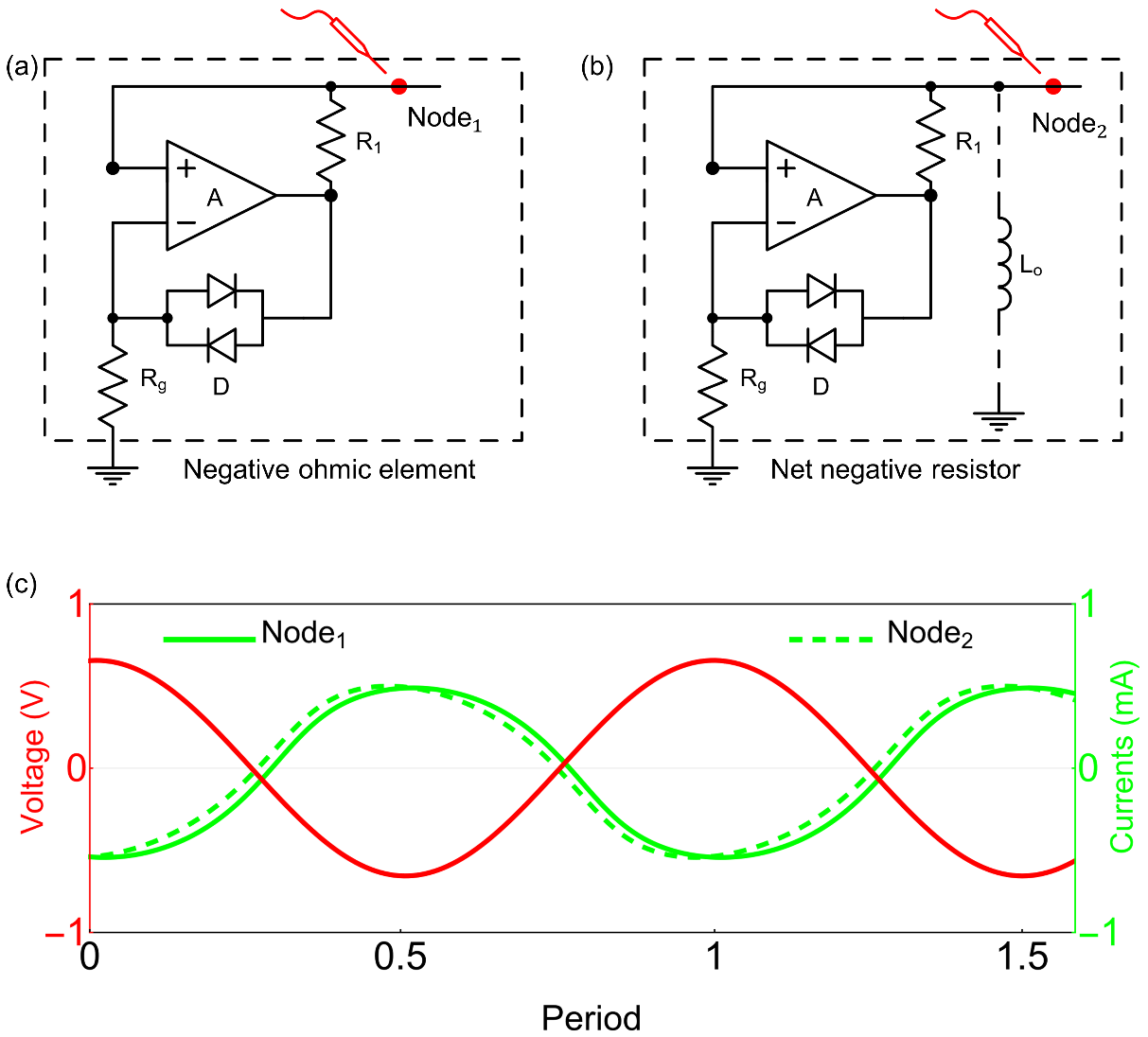


**Figure S15.** (a) The circuit shows the implementation of a negative resistor with normal resistors (R), diodes (D) and the op-amp (A). The phase difference between the current and voltage at node_1_ for an ideal negative ohmic resistor should be $\pi$. However, the unavoidable parasitic capacitance renders this phase difference to deviate from $\pi$. The deviation can be compensated by an offset inductor $L_{o}$ as shown in (b). As sketched in (c), the phase difference between the current at node_1_ (solid green line) and the applied voltage (red line) slightly deviates from $\pi$. With the offset inductor $L_{o}$, the phase difference between the current (dashed green line) at node_2_ and the applied voltage (red line) becomes $\pi$ as can be seen from the crossings of those two curves at zero.

The effect of this offset inductor $L_{o}$ is investigated experimentally by constructing a LC resonance circuit as shown in Fig. S8(a). The value of $L_{o}$ depends on two dominant parameters. One is the resistor *R*_1_ which changes the gain profile and the other is the voltage at node_1_. In the experiment, we vary the resistance of *R*_1_ to keep $V_{B}\approx1V$ and the range of *R*_1_ is measured to be [400, 500]$\Omega$. Here we choose five representative values of *R*_1_ = {400, 425, 450, 475, 500}$\Omega$ and then measure the resonance frequency of the LC circuit as a function of the applied voltage. Here the voltage can be changed by varying the loaded resistance *R* as shown in Fig. S8(d).

Figure S16(a) shows the measured steady resonance frequency as functions of voltage for different *R*_1_. The resonance frequency $f$ satisfies

$$\begin{aligned} f={[C\left( L+\Delta_{L} \right)]}^{-\frac{1}{2}},\#\left( S34 \right) \end{aligned}$$

where the capacitance *C* and inductance *L* can be measured experimentally, and $\Delta_{L}$ labels the shift induced by the offset inductor $L_{o}$ with different *R*_1_ and voltage. As shown in Fig. S14, the capacitance is almost independent of the working frequency and voltage with the measured value $C=22.2 \mathrm{nF}$. Figure S16(b) shows the measured inductance *L* as a function of voltage. Here the upper edge and lower edge of the cyan strip represent the inductances measured at 71.7 kHz and 71.9 kHz, respectively, and the black line shows the average value. Once again, we can see the inductance depends weakly on the working frequency, and for simplicity, we take the average value (black line) as the measured inductance. With Eq. (S$34$), we can obtain $\Delta_{L}$ as functions of the applied voltage for different *R*_1_ as shown in Fig. S16(c).

When the voltage is around 0.83V (the values of *V_A_* for the EPs in Fig. S12(a) and (e), $\Delta_{L}$ is measured to be 2.07$\pm0.9$ $\mu H$, which is consistent with the shift of $L_{A}$ by 2.2$\mu H$. When the voltage is around 1.0V (the values of *V_A_* for the EX in Fig. S12(c, g) ), $\Delta_{L}$ is measured to be 1.0$\pm0.9$ $\mu H$, which is consistent with the shift of $L_{A}$ by 1.4$\mu H$. Thus the shift of measured $L_{A}$ due to the parasitic capacitance to match the theoretical model is consistent with the measured value. We want to point out that here $\Delta_{L}$ we are dealing with is already pretty small (less than 1%).


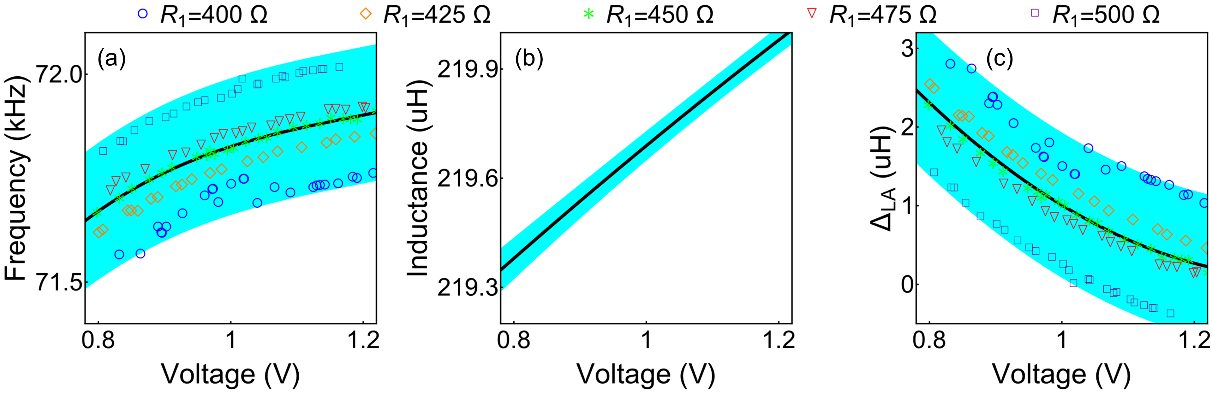


**Figure S16.** Dependence of the resonance frequency (a), the measured inductance *L* (b) and the shift of inductance $\Delta_{L}$ (c) on the voltage with different $R_{1}$. In these experiments, we use the circuit in Fig. S8(a), and the increase of voltage is provided by the increasing resistance *R*. The black solid lines denote the average for eye guiding. $R_{g}=5.1 k\Omega$ and $C=22.2 nF$ are used in the experiments

The effective inductance $L_{A}$ can also be determined self-consistently. As can be seen from Eq. (S$29$), there is a one to one relation between $L_{A}$ (assuming $L_{B}$ is constant since we fix $V_{B}\approx1V$) and $\left| V_{A}/V_{B} \right|$ for each steady state. Once we have measured $\left| V_{A}/V_{B} \right|$ experimentally, we can calculate the corresponding $L_{A}$ through this relation. Figure S17 shows the steady state frequencies versus effective inductance $L_{A}$ for the EPs and EX. We can see the measured data agrees almost perfectly with the numerical results from the Kirchoff’s equations.


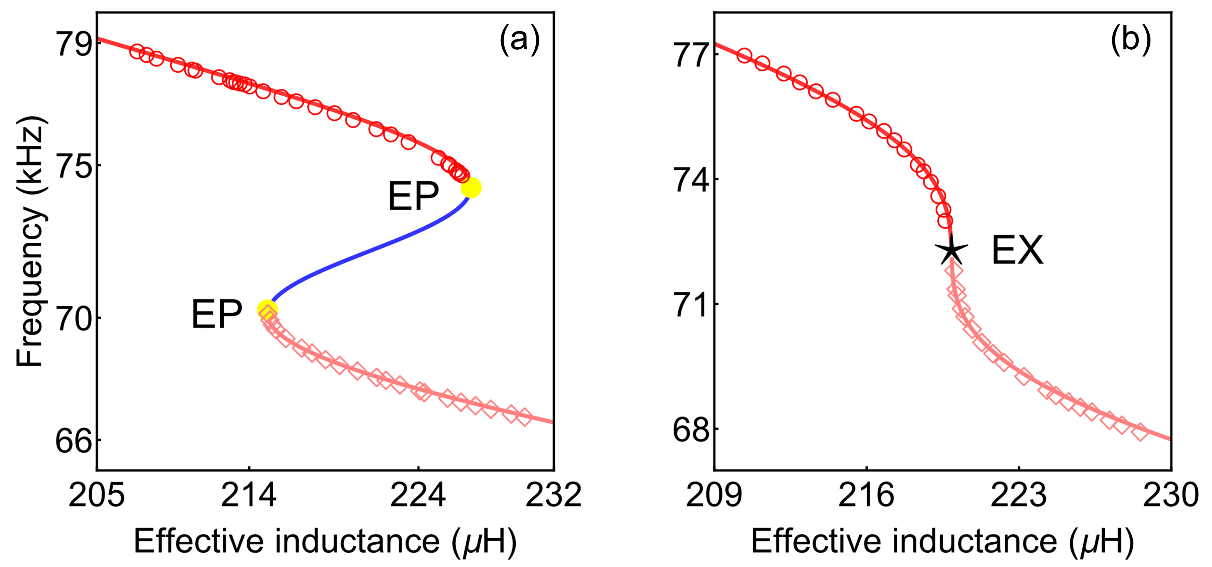


**Figure S17.** The steady-state frequency as a function of effective inductance at$R_{2}$=1$k\Omega$ (a) and $R_{2}$=708$\Omega$ (b).

**REFERENCES**

1. Cerjan A, Stone AD. Steady-state ab initio theory of lasers with injected signals. *Phys Rev A* 2014; **90**: 013840.

2. Assawaworrarit S, Yu X, Fan S. Robust wireless power transfer using a nonlinear parity–time-symmetric circuit. *Nature* 2017; **546**: 387–390.

3. Wang H, Assawaworrarit S, Fan S. Dynamics for encircling an exceptional point in a nonlinear non-Hermitian system. *Opt Lett* 2019; **44**: 638.

4. Tang W, Jiang X, Ding K, *et al.* Exceptional nexus with a hybrid topological invariant. *Science* 2020; **370**: 1077–1080.

5. Mandal I, Bergholtz EJ. Symmetry and Higher-Order Exceptional Points. *Phys Rev Lett* 2021; **127**: 186601.

6. Ding K, Ma G, Xiao M, *et al.* Emergence, coalescence, and topological properties of multiple exceptional points and their experimental realization. *Phys Rev X* 2016; **6**: 1–13.

7. Zhong Q, Kou J, Özdemir ŞK, *et al.* Hierarchical Construction of Higher-Order Exceptional Points. *Phys Rev Lett* 2020; **125**: 203602.

8. Delplace P, Yoshida T, Hatsugai Y. Symmetry-protected higher-order exceptional points and their topological characterization. *Phys Rev Lett* 2021; **127**: 186602.

9. Hodaei H, Hassan AU, Wittek S, *et al.* Enhanced sensitivity at higher-order exceptional points. *Nature* 2017; **548**: 187–191.

10. Bender CM, Mannheim PD. PT symmetry and necessary and sufficient conditions for the reality of energy eigenvalues. *Phys Lett A* 2010; **374**: 1616–1620.

11. Lee S-Y, Ryu J-W, Shim J-B, *et al.* Divergent Petermann factor of interacting resonances in a stadium-shaped microcavity. *Phys Rev A* 2008; **78**: 015805.

12. https://www.analog.com/en/design-center/design-tools-and-calculators/ltspice-simulator.html

13. Adapted from the https://www.bourns.com/data/global/pdfs/ap_proc.pdf
